# Dissemination of *Staphylococcus epidermidis* in Swedish bovine dairy herds: minimal overlap with human isolates

**DOI:** 10.3389/fmicb.2025.1512461

**Published:** 2025-02-07

**Authors:** Karin Persson Waller, Mattias Myrenås, Hyeyoung Kim, Micael Widerström, Tor Monsen, Stefan Börjesson, Emma Östlund, Wonhee Cha

**Affiliations:** ^1^Department of Animal Health and Antimicrobial Strategies, Swedish Veterinary Agency (SVA), Uppsala, Sweden; ^2^Department of Epidemiology, Surveillance and Risk Assessment, Swedish Veterinary Agency (SVA), Uppsala, Sweden; ^3^Department of Clinical Microbiology, Umeå University, Umeå, Sweden; ^4^School of Health Science, Örebro University, Örebro, Sweden; ^5^Department of Food Safety and Animal Health Research, Norwegian Veterinary Institute, Ås, Norway; ^6^Department of Microbiology, Swedish Veterinary Agency (SVA), Uppsala, Sweden

**Keywords:** mastitis, dairy cows, WGS—whole-genome sequencing, antimicrobial resistance (AMR) genes, virulence factors (VF), *Staphylococcus epidermidis*, one health (OH)-approach

## Abstract

Knowledge of zoonotic links between bovine and human isolates of *Staphylococcus epidermidis* remains limited. The primary aim of this study was to assess the genetic relatedness of *S. epidermidis* isolates from bovine subclinical mastitis (SCM), bovine milk filters, healthy dairy farmers or farm personnel, and human hospital patients in Sweden, and to detect and compare genes encoding antimicrobial resistance (AMR) and virulence factors. A secondary aim was to explore the epidemiology of bovine *S. epidermidis* intramammary infections (IMI) by examining associations between genotypes and geographic location, persistence of IMI, severity of inflammatory response, and the persistence of *S. epidermidis* strains over time. A total of 283 *S. epidermidis* isolates were analyzed using whole genome sequencing (WGS): 128 SCM milk isolates, 55 milk filter isolates, 13 farmer/personnel isolates, and 87 human patient isolates. Sixty unique sequence types (STs) of *S. epidermidis* were identified. ST99, ST100, and ST570 were the most common among bovine isolates, collectively accounting for 49% (63/128) of the milk isolates and detected on multiple farms, while ST2 and ST215 were the most prevalent among human isolates. Only four STs (ST59, ST73, ST184, and ST218), representing a total of 13 isolates, were found in both bovine and human samples. Genes conferring AMR were more frequently identified in human patient isolates compared to bovine isolates. However, penicillin resistance, identified by presence of the *blaZ* gene, was detected in 42% of bovine *S. epidermidis* isolates. The average number of potential virulence factors (pVF) per isolate was 23.8 with 23.1 in milk isolates, 23.4 in milk filter isolates, 23.0 in farmer/personnel isolates, and 25.2 in human patient isolates. There was some variation in the total number of pVFs and the presence of specific pVFs or functional groups of pVFs between sample types and STs. In conclusion, the results indicate that the overlap of STs and AMR genes between human and bovine samples was minimal. However, the persistence of certain STs across multiple dairy farms suggests inter-farm transmission. This study provides new insights into the epidemiology of bovine *S. epidermidis* IMI, with implications for the control of these infections.

## 1 Introduction

*Staphylococcus epidermidis* is a bacteria found in the normal flora of the skin and mucosa of humans and animals but is also an opportunistic pathogen (Severn and Horswill, 2023). In humans, *S. epidermidis* has emerged as a significant cause of healthcare-associated infections frequently associated with blood stream infections and postoperative infection, mainly related to the increased use of indwelling medical devices (Becker et al., 2014). In bovine dairy cows, these bacteria are a common cause of intra-mammary infection (IMI), mostly resulting in subclinical mastitis (Persson Waller et al., 2011; Nyman et al., 2018; De Buck et al., 2021). In Sweden, the prevalence of *S. epidermidis* IMI is markedly higher among dairy cows compared to other countries (Nyman et al., 2018), but the reasons for this remain unknown. In addition, there is a general lack of knowledge about the epidemiology of *S. epidermidis c*onnected to IMI, due the limited number of studies on genotypic variation of these bacteria.

It is likely that *S. epidermidis* strains can spread between cattle and humans, as it is known that *Staphylococci* can transfer from animals to humans. In particular, transfer of methicillin-resistant *S. aureus* (MRSA) from pigs to humans is well-described (Abdullahi et al., 2023). Transfer can also occur from humans to cattle, and a recent case study showed spread of MRSA from a farmer to dairy cows in a herd (Ericsson Unnerstad et al., 2018). In the case of *S. epidermidis*, the same genotype, as evaluated by pulsed-field gel electrophoresis, was found both on the hands of healthy humans and in milk from dairy cows in farms in Sweden (Thorberg et al., 2006). Similar findings, but of another genotype, were also made in a study from the Czech Republic (Jaglic et al., 2010).

Genes encoding for antimicrobial resistance (AMR) may also spread between bovine and human strains of *S. epidermidis*. Multidrug resistance is common among *S. epidermidis* isolates from patients in hospitals in several countries, including Sweden (Monsen et al., 1999; Lee et al., 2018; Widerström et al., 2016; Månsson et al., 2021), while it is less frequent in isolates from healthy humans (Widerström et al., 2011a). Although multidrug resistance is rarely observed in the isolates from Swedish bovine IMI (Persson Waller et al., 2011; Duse et al., 2021), beta-lactamase production has been described in ~40% of *S. epidermidis* isolates from bovine subclinical mastitis (Persson Waller et al., 2011; Nyman et al., 2018). These findings warrant investigations of AMR genes in bovine *S. epidermidis* isolates and further comparison of AMR genes in bovine and human isolates.

The presence of genes encoding virulence factors, indicating potential virulence, varies among bovine and human isolates of *S. epidermidis* (Månsson et al., 2021; Naushad et al., 2019). Such variation may, at least partly, explain why the clinical outcome of *S. epidermidis* IMI varies between studies. As is the case for AMR genes, there is also a potential for spread of virulence genes between isolates of *S. epidermidis* (Meric et al., 2018). Thus, it is possible that virulence genes may spread between bovine and human strains with possible implications for clinical disease, as has been described in pig farms (Lee et al., 2023).

Currently, knowledge regarding zoonotic links between bovine and human isolates of *S. epidermidis* is limited. Therefore, the primary aim of the present study was to assess the genetic relatedness of isolates, as well as to detect and compare genes encoding AMR or virulence, from bovine cases of subclinical mastitis, bovine milk filters, healthy dairy farmers or farm personnel, and human hospital patients. A secondary aim was to study the epidemiology of bovine *S. epidermidis* IMI by analyzing associations between genotypes, geographic location, and movement of cows between farms. When feasible, associations between genotypes, persistent IMI, and the severity of inflammatory response were also examined.

## 2 Materials and methods

**Figure 1:**
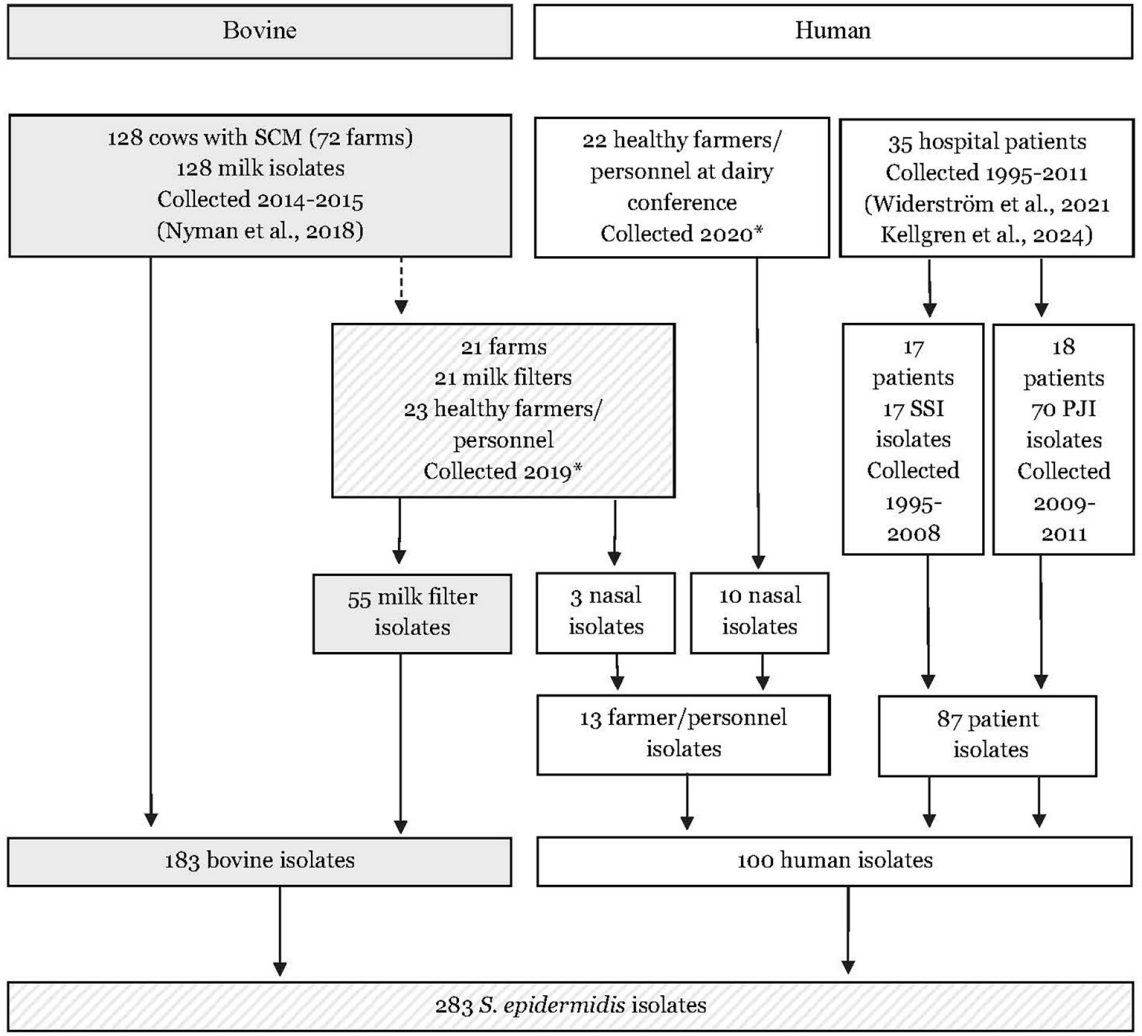
Flow chart describing the origin of 283 bovine and human isolates of *Staphylococcus epidermidis* included in the study. SCM, subclinical mastitis; PJI, prosthetic joint infection; SSI, surgical site infections. * Samples and isolated were collected in the present study.

In the present study, sequencing data from a total of 183 bovine *S. epidermidis* isolates and 100 human *S. epidermidis* isolates were included. The study material is presented in Figure 1 and explained in detail below.

### 2.1 Bovine and human isolates

In the present study, *S. epidermidis* milk isolates collected during a study on bovine subclinical mastitis in Sweden (2014–2015) were included (Nyman et al., 2018). In the original study, 206 isolates were identified as *S. epidermidis* using matrix-assisted laser desorption ionization-time of flight mass spectrometry (MALDI-TOF MS; Bruker Daltronics, Bremen, Germany). All isolates were stored at −80°C in trypticase soy broth containing 15% glycerol. For the present study, 128 isolates were selected. These isolates originated from 128 cows across 72 farms located in 18 of Sweden's 21 counties. To study between-farm variation, one isolate from each farm (*n* = 72) was included, selecting the first identified isolate when multiple isolates were available from the same farm. To study within-farm variation, only farms with isolates from at least three cows were included (*n* = 15). For these farms, 3–10 isolates were selected per farm, with only the first identified isolate included if multiple isolates were available from the same cow.

For the current study, 72 farms selected from 2014 to 2015 were contacted via postal mail in the end of 2019, and 21 (29.2%) of them chose to participate in a follow-up study. The farmers were asked to collect the milk filter placed at the entrance valve of the bulk milk tank directly after one morning or afternoon milking of all lactating cows. The filters were placed in sterile plastic bags, stored with ice packs in a styrofoam box and sent over-night to the Swedish Veterinary Agency (SVA, Uppsala, Sweden). At the laboratory, each filter was placed in 200 mL Müller Hinton broth with 6.5% NaCl (SVA) in a stomacher bag and was blended for 30 s using a Stomacher paddle blender (Seward Ltd., Worthing, UK) at 230 rpm and thereafter divided into two aliquots. One aliquot was diluted 1:100 with Müller Hinton broth with 6.5% NaCl, from which 50 μL was spread on CHROMagar™ Staphylococcus (Paris, France) and incubated for 18 h at 37°C. From the other aliquot, 45 mL was cultured f or 18 h in a tube at 37°C and diluted 1:10 with Müller Hinton broth with 6.5% NaCl. Then, 50 μL of the dilution was cultured on CHROMagar^TM^ as above. From each agar plate, a maximum of 10 suspected staphylococci colonies were subcultured on 5% (v/v) horse blood agar plates (SVA; blood agar base number 2 Oxoid) overnight. Species identification was made using MALDI-TOF MS. Altogether, 55 *S. epidermidis* isolates (1–5 isolates/milk filter and farm) were obtained from 17 (81%) of 21 milk filters from respective farms. The isolates were stored at −80°C in trypticase soy broth containing 15% glycerol until further analysis.

The farmers and farm personnel of 21 farms were also asked to take one nasal sample from both nostrils using a Transystem^TM^ Sterile transport swab for aerobes and anaerobes (Copan S.p.A, Brescia, Italy) according to instructions provided (Ethical permission 2019-01288, Swedish Ethical Review Authority). The samples were sent chilled to SVA together with the milk filters. A total of 23 (3 from 1 farm and 1 from 20 farms) nasal samples from 23 persons were received and forwarded to the Department of Clinical Microbiology, Umeå University Hospital (Umeå, Sweden, accreditation no 1397). The swabs were streaked on blood agar plates (Aerobic Columbia horse blood agar (Neogen blood agar base; Neogen Europe Limited, Lancashire, UK) supplemented with 5% (vol/vol) horse blood (Håtunalab AB, Bro, Sweden) prior to incubation at 37°C over night (20–24 h) before examination. *S. epidermidis* was identified using MALDI-TOF MS. Growth of *S. epidermidis* was detected in 3 (13%) out of 23 nasal samples resulting in three isolates. The isolates were stored at −80°C until further examination.

Additional samples from farmers or farm personnel were collected at a conference for Swedish dairy farmers (Växa, Örebro) in the beginning of 2020. A total of 22 dairy farmers or farm personnel volunteered to take nasal samples as described above. These nasal samples were stored with ice packs in a cooling box before being sent to the Department of Clinical Microbiology, Umeå, for culturing as described above. In total 10 *S. epidermidis* isolates were identified from 8 (36.4%) of 22 nasal samples. The isolates were stored at −80°C until further examination.

Finally, sequencing data from 87 human clinical isolates of *S. epidermidis*, obtained from Swedish hospitals between 1995 and 2011, were accessible through two other studies (Widerström et al., 2022; Kellgren et al., 2024). These isolates originated in 35 patients, with 1–8 isolates per patient. Among them, 70 isolates were retrieved from 60 cultures of 18 patients diagnosed with prosthetic joint infections (PJI) between 2008 and 2011, while 17 isolates originated from 17 patients with surgical site infections (SSI) occurring from 1995 to 2008. These clinical isolates were selected to represent two previously described highly antibiotic-resistant hospital genetic lineages with a distribution among multiple hospitals in Sweden (Widerström et al., 2011b).

### 2.2 Whole-genome sequencing, genome assembly, and annotation

The bovine isolates and the human isolates collected from farmers and farm personnel (*n* = 13) were thawed and cultured on 5% bovine blood agar with 0.05% esculin (Swedish Veterinary Agency, SVA) for 16–24 h at 37°C. All cultures were stored at 4°C until further processing and DNA extraction was performed using Qiagen EZ1 DNA Tissue Kit (Qiagen, Halden, Germany). Whole genome sequencing (WGS) was performed at Clinical Genomics Stockholm, SciLifeLab (Solna, Sweden) using Nextera Library preparation (Illumina, Foster City, United States) and paired-end sequencing (2 × 150 bp) on an Illumina Novaseq 6000 instrument. The unprocessed sequence data for each sample were quality checked using FastQC v0.11.9 (Andrews, 2010), trimmed using Trimmomatic v0.39 (Bolger et al., 2014), and assembled using SPAdes v3.14.0 (Prjibelski et al., 2020). Assemblies were then error-corrected using Pilon v1.23 (Walker et al., 2014). Sequence assemblies were annotated using Prokka v1.12 (Seemann, 2014). Details on the analyses can be found in Supplementary Table 1.

Whole-genome sequencing of the 70 human clinical isolates from PJI was performed as described previously (Widerström et al., 2022), while WGS of the 17 human isolates from cases of SSI was performed on the Illumina HiSeq 2000 platform (SciLifeLab, Uppsala, Sweden), following standard procedures for DNA preparation, library construction, and genome sequencing as per the manufacturer's instructions. The analysis of the sequence data was performed as described above.

### 2.3 Bioinformatic analyses

A 7-locus multilocus sequence typing (7-MLST) of *S. epidermidis* was performed using the scheme (Thomas et al., 2007) available at PubMLST (*S. epidermidis*). Using the 7-MLST profiles, a minimum spanning tree was constructed using SeqSphere+ v8.3 (Ridom, Würzburg, Germany) to show the distribution and genomic relationships between the strains.

Roary v3.12.0 (Page et al., 2015) was used to compute the core genomes of the strains with parameters of -i (blastp of 90%) and -e to create a multiFASTA alignment of core genes, from which a tree was generated using FastTree v2.1.10 (Price et al., 2010) with -gtr model parameter. The visualization of Roary result was done using Phandango v1.3.1 (Hadfield et al., 2018).

An *ad hoc* cgMLST scheme was created in SeqSphere+ using the annotated genome GenBank accession numbers NZ_HG813242.1 and NZ_CP035643.1 as seed genome and penetration genome, respectively, and excluding the plasmid genomes NZ_HG813246.1, NZ_HG813245.1, NZ_HG813244.1, and NZ_HG813243.1, resulting in a cgMLST scheme consisting of 1,840 genes. This scheme was used to analyze the assembled contigs created in the project and to calculate the phylogenetic distance using the Neighbor-Joining Tree method (Saitou and Nei, 1987) included in SeqSphere+. A total of 919 alleles were missing in at least one sample and were handled using pairwise exclusion of missing alleles. No sample had missing values for more than 6% of the alleles. The resulting trees were visualized in iTol v6 (Letunic and Bork, 2016). The cgMLST data for samples with ST99, ST100, and ST570 was also used for creating minimum spanning trees in SeqSphere+.

### 2.4 *In-silico* analysis of antimicrobial resistance and virulence factors

We used the ResFinder database (https://cge.cbs.dtu.dk/services/ResFinder, accessed on April 16th, 2020) to search for antimicrobial resistance genes using Antimicrobial Resistance Identification By Assembly (ARIBA) with the settings ≥90% identity and 100% coverage (Hunt et al., 2017). To look for point mutations in the genes *dfrC, gyrA, grlA, parB, parC, pare*, and *rpoB*, the genes were extracted, translated, and manually compared to *S. epidermidis* ATCC 12228 using CLC Main Workbench v21.0.5 (https://digitalinsights.qiagen.com). We also looked for the presence of additional genes ACME-*arcA, bhp, embp, fdh, qacA/B*, and *ses*I as described in a previous study by Månsson et al. (2021).

The identification of potential virulence factors (pVFs) was conducted as previously described (Naushad et al., 2019), involving calculation of H scores and reciprocal blast searches (Ward and Moreno-Hagelsieb, 2014) between the pVFs and genes in the VF database defined in this study (Supplementary Table 2).

### 2.5 Herd and cow data

The geographical location of each herd included in the study was retrieved from Geodata portal (www.geodata.se). The movement data of each cow in the study was obtained from the national cattle database at the Swedish Board of Agriculture, which encompasses all movement events at the individual level, including birth, death, export, as well as temporary movements (Nöremark et al., 2009). A time period from September 2011 to May 2015 was selected for the analysis based on the estimated impact of cattle movements on the potential introduction of mastitis-associated pathogens.

Information on the California Mastitis Test (CMT; Schalm and Noorlander, 1957) scores (ranges 1–5; 1 = no reaction, 2 = trace, 3 = slight, 4 = moderate, 5 = strong reaction) in quarter milk samples, as well as data on udder quarters with persistent IMI (defined as having the same NAS species in the same quarter at two consecutive samplings, on average 32 days apart) were obtained from the study by Nyman et al. (2018).

### 2.6 Statistical analyses

**Figure 2:**
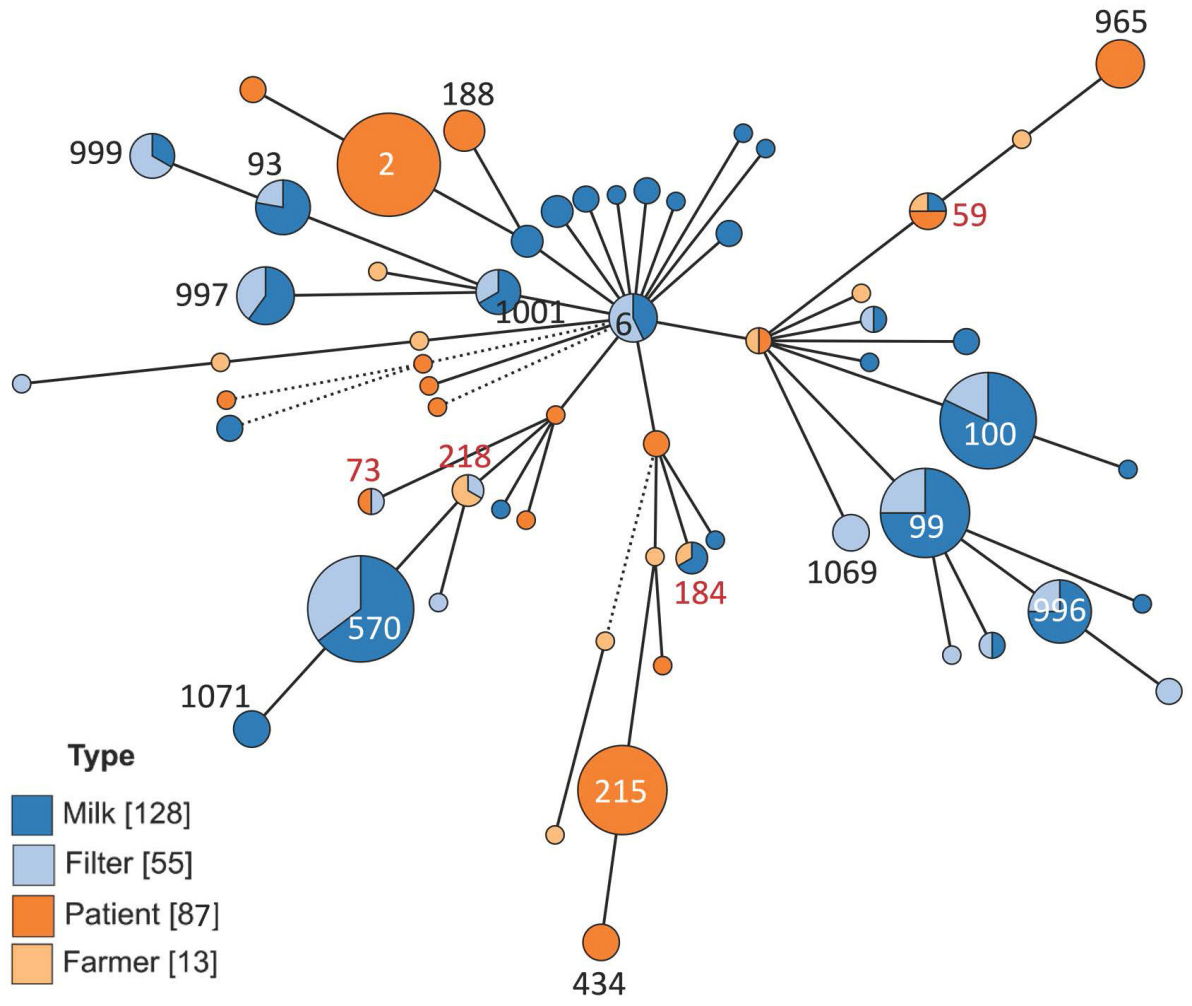
Minimum spanning tree showing the distribution of multi-locus sequence types of 283 isolates of *Staphylococcus epidermidis*. The isolates came from bovine milk, bovine milk filters, healthy dairy farmers/personnel, or human hospital patients. The size of the circles indicates the number of isolates. Solid short lines indicate difference in one allele out of seven. Solid longer lines two alleles difference, dashed lines indicate more than two alleles difference.

**Figure 3:**
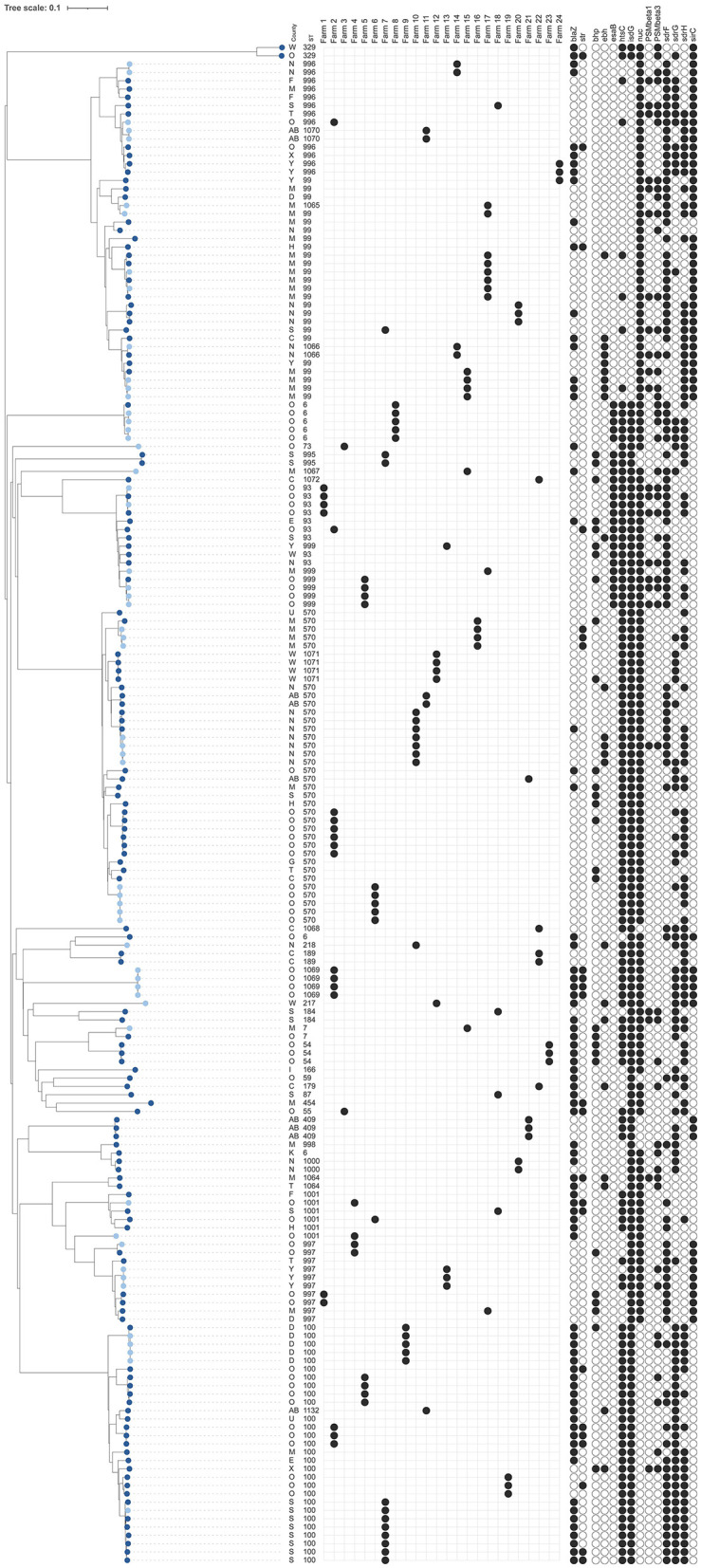
Dendrogram of core-genome multi-locus sequence typing of 183 bovine isolates of *Staphylococcus epidermidis* [128 milk isolates (dark blue) and 55 bovine milk filter isolates (light blue)]. Information on county, sequence types (ST), distribution within 24 farms with at least three milk isolates or both milk and milk filter isolates, presence of antimicrobial resistance genes [*blaZ, str*pS194 (*str*)], and detection of genes for potential virulence factors that were present in 5%−95% of all isolates (see Supplementary Table 1 for key) are shown. The scale is a unit-less ratio of core-genome multi-locus sequence typing allele similarities.

Logistic regression was used to assess the associations between genotypes, farms, and geographical locations, with the ST as the outcome variable. Analyses were performed for each ST that included at least five milk isolates. The distribution of all STs by herd and county was also manually assessed and presented in Tables 1, 2 and Figures 2, 3. Animal movements between herds and their associations with prevalent STs and AMR genes at the herd level were analyzed in R v.4.1.0 (R Core Team, 2021), incorporating temporal information into the network structure. Using the EpiContactTrace package (Nöremark and Widgren, 2014), herds were represented as nodes, and cattle movement events during the study period were represented as edges. Temporal sequences of direct and indirect contacts between herds were considered in calculating the network measure contact chain, enabling backward and forward tracing of movements over time. Associations between STs and persistent IMI were examined using Fisher's exact test, while associations with CMT scores were examined using a cumulative link mixed model for ordinal regression, with herd included as a random effect.

**Table 1:** Numbers (N) of *Staphylococcus epidermidis* isolates from bovine milk, bovine milk filters, healthy dairy farmers/personnel, and human hospital patients distributed per multi-locus sequence type (ST).

**ST**	**Milk isolates (farms) *N* = 128 (72)**	**Milk filter isolates (farms) *N* = 55 (17)**	**Farmer/personnel isolates (farms) *N* = 13 (12)**	**Patient isolates (humans) *N* = 87 (35)**	**All isolates *N* = 283**
2	0	0	0	**32 (13)**	**32**
5	0	0	1 (1)	1 (1)	2
6	3 (3)	4 (1)	0	0	7
7	1 (1)	1 (1)	0	0	2
14	0	0	1 (1)	0	1
22	0	0	0	2 (2)	2
32	0	0	1 (1)	0	1
54	3 (1)	0	0	0	3
55	1 (1)	0	0	0	1
59	1 (1)	0	1 (1)	2 (2)	4
73	0	1 (1)	0	1 (1)	2
81	0	0	1 (1)	0	1
86	0	0	0	1 (1)	1
87	1 (1)	0	0	0	1
89	0	0	0	2 (1)	2
93	7 (6)	2 (2)	0	0	9
99	**18 (13)**	**6 (2)**	0	0	24
100	**23 (10)**	5 (2)	0	0	28
152	0	0	0	1 (1)	1
153	0	0	1 (1)	0	1
166	1 (1)	0	0	0	1
179	1 (1)	0	0	0	1
184	2 (2)	0	1 (1)	0	3
188	0	0	0	5 (1)	5
189	2 (1)	0	0	0	2
215	0	0	0	**24 (15)**	24
217	0	1 (1)	0	0	1
218	0	1 (1)	**2 (1)**	0	3
225	0	0	0	1 (1)	1
329	2 (2)	0	0	0	2
355	0	0	1 (1)	0	1
409	3 (1)	0	0	0	3
434	0	0	0	4 (1)	4
454	1 (1)	0	0	0	1
487	0	0	1 (1)	0	1
570	**22 (14)**	**12 (3)**	0	0	**34**
723	0	0	0	1 (1)	1
961	0	0	0	1 (1)	1
962	0	0	0	1 (1)	1
964	0	0	0	1 (1)	1
965	0	0	0	7 (1)	7
995	2 (1)	0	0	0	2
996	9 (8)	3 (2)	0	0	12
997	6 (5)	4 (2)	0	0	10
998	1 (1)	0	0	0	1
999	2 (2)	4 (2)	0	0	6
1,000	2 (2)	0	0	0	2
1,001	4 (4)	2 (1)	0	0	6
1,002	0	0	1 (1)	0	1
1,003	0	0	1 (1)	0	1
1,064	2 (2)	0	0	0	2
1,065	0	1 (1)	0	0	1
1,066	1 (1)	1 (1)	0	0	2
1,067	0	1 (1)	0	0	1
1,068	1 (1)	0	0	0	1
1,069	0	4 (1)	0	0	4
1,070	0	2 (1)	0	0	2
1,071	4 (1)	0	0	0	4
1,072	1 (1)	0	0	0	1
1,132	1 (1)	0	0	0	1

The numbers per STs with at least 10% of the isolates within each group are highlighted in bold. The numbers of farms or humans from which the isolates originated are given in brackets.

**Table 2:** Within-farm comparisons of sequence types (STs) of *Staphylococcus epidermidis* isolates isolated from bovine milk or milk filters.

**Farm**	**Milk isolates**			**Milk filter isolates (comparison with milk isolates collected 4–5 years earlier at the same farm)**		
	**Number of isolates**	**ST (number of isolates)**	**The same ST in** >**1 cow**^1^ **(Yes/No)**	**Number of isolates**	**ST (number of isolates)**	**The same ST as in milk (Yes/No)**
1	4	93 (2) 997 (2)	Yes	2	93	Yes
2	10	93 (1) 100 (3) 570 (6)	Yes	5	996 (1) 1,069 (4)	No
3	1	55	–^2^	1	73	No
4	1	997	–	3	997 (1) 1,001 (2)	Yes
5	5	100 (4) 999 (1)	Yes	3	999 (3)	Yes
6	1	1,001	–	5	570 (5)	No
7	10	99 (1) 100 (7) 995 (2)	Yes	1	100	Yes
8	1	6	–	4	6 (4)	Yes
9	1	100	–	4	100 (4)	Yes
10	3	570 (3)	Yes	5	218 (1) 570 (4)	Yes
11	3	570 (2) 1,132 (1)	Yes	2	1,070 (2)	No
12	4	1,071 (4)	Yes	1	217	No
13	1	999	–	3	997 (3)	No
14	1	1,066	–	3	996 (2) 1,066 (1)	Yes
15	1	99	–	5	7 (1) 99 (3) 1,067 (1)	Yes
16	1	570	–	3	570 (3)	Yes
17	5	99 (4) 997 (1)	Yes	5	99 (3) 999 (1) 1,065 (1)	Yes
18	4	87 (1) 184 (1) 996 (1) 1,001 (1)	No	–	–	–
19	3	100 (3)	Yes	–	–	–
20	5	99 (3) 1,000 (2)	Yes	–	–	–
21	4	409 (3) 570 (1)	Yes	–	–	–
22	5	179 (1) 189 (2) 1,068 (1) 1,072 (1)	Yes	–	–	–
23	3	54 (3)	Yes	–	–	–
24	3	99 (1) 996 (2)	Yes	–	–	–

Fifteen farms had at least three milk isolates collected between 2014 and 2015 from cows with subclinical mastitis. Seventeen farms had at least one milk isolate collected between 2014 and 2015 from cows with subclinical mastitis, and at least one milk filter isolate collected in 2019.

^1^ Based on comparison within farms with at least 3 milk isolates.

^2^ Not applicable.

Differences in the total number of genes and point mutations conferring AMR and virulence (pVFs) between sample types or STs, were assessed using the Wilcoxon rank-sum test. The distributions of individual pVF between sample types were evaluated using either chi-squared test or Fisher's exact test, depending on the sample size. Associations between STs and presence or absence of genes for AMR or pVFs were analyzed by mixed-effect logistic regression, with patient or herd added as a random effect. For milk samples, associations between the number of pVFs (total and by functional group) and persistent IMI, as well as increased CMT score (i.e., CMT3–5), were examined using mixed-effect logistic regression and mixed-effect ordinal logistic regression models, respectively, with herd as the random effect, as previously described (Persson Waller et al., 2023). All statistical analysis, except for network analysis, was performed using R v4.4.0 (R Core Team, 2021). A *P*-value of < 0.05 was used as the cutoff for statistical significance in all analyses.

## 3 Results

### 3.1 Genetic diversity of *S. epidermidis*

**Figure 4:**
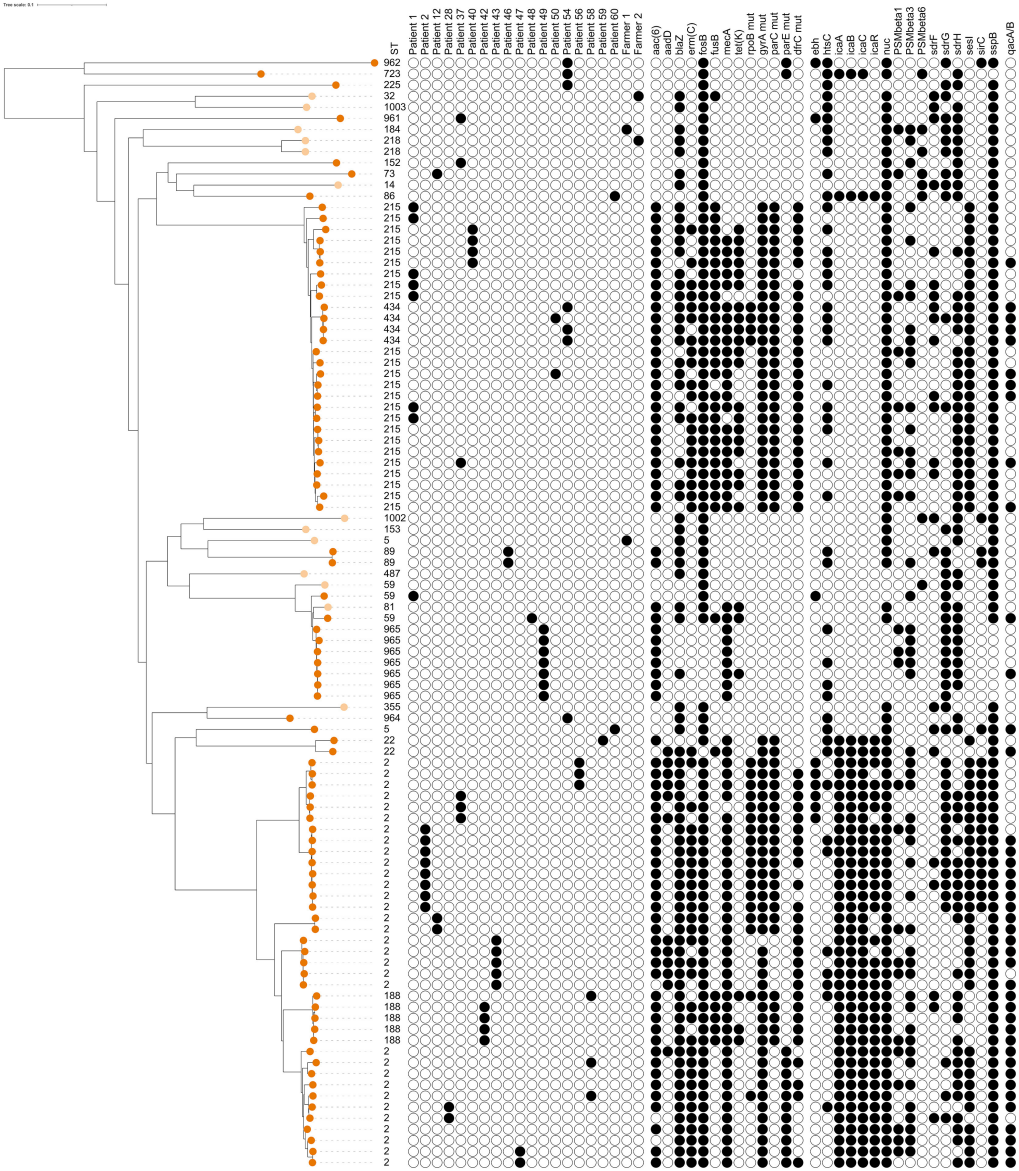
Dendrogram of core-genome multi-locus sequence typing of 100 human isolates of *Staphylococcus epidermidis* [13 isolates from healthy farmers/personnel (light orange) and 87 isolates from human hospital patients (dark orange)]. Information on sequence types (ST), distribution within 18 patients and 2 farmers with at least 2 isolates, presence of antimicrobial resistance genes or point mutations, and detection of genes for potential virulence factors that were present in 5%−95% of all isolates (see Supplementary Table 1 for key). The scale is a unit-less ratio of core-genome multi-locus sequence typing allele similarities.

The results on genetic diversity of 283 investigated isolates are summarized in Table 1, Figures 2–4, and Supplementary Figure 1. In total, 60 unique sequence types (STs) were identified (Table 1). Among those, 19 were identified as new and were deposited in the PubMLST database: ST995 to ST1003 (id 41591–41599), ST1064 to ST1072 (id 41672–41680), and ST1132 (id 41748). Overall, ST570 (*n* = 34) and ST2 (*n* = 32) were most prevalent, followed by ST100 (*n* = 28), ST99 (*n* = 24), and ST215 (*n* = 24). Together, these five STs constituted 50.2% of the isolates. The distribution of STs among the four sample types is described below.

#### 3.1.1 Genetic diversity of bovine *S. epidermidis* and associations between genotypes and geography, cattle movements, persistent IMI and CMT

As already mentioned, 128 *S. epidermidis* milk isolates were included (Table 1, Figure 3), among which a total of 30 STs were identified, 13 of which were new. The most common STs were ST99, ST100, and ST570, together constituting 49% (63 out of 128) of the milk isolates. These three STs were also the most common STs found among the 55 milk filter isolates collected in 2019 (Table 1). The general diversity index (DI), estimated as number of STs/number of isolates, was similar among milk isolates [DI = 0.910 (CI = 0.886–0.935); 30 STs/128 isolates] and milk filter isolates [DI = 0.919 (CI = 0.882–0.955); 18 STs/55 isolates].

Out of 72 farms contributing milk isolates, ST99, ST100, and ST570 were identified in 13, 10, and 14 herds, respectively (Table 1). Among the remaining 14 STs having at least two isolates, 9 STs were detected in more than one herd (2–8 herds/ST). In Table 2, comparisons of bovine isolates within farms (milk isolates) and within farms over time (milk isolates vs. milk filter isolates) are presented. In 14 of 15 farms (93%) with at least three milk isolates, the same ST was found in more than one cow (2–7 cows/herd). Among the 11 STs that had this pattern, ST100 was found in four farms and ST570 in three farms. Moreover, in 11 out of 17 farms (65%) with isolates from both milk and milk filters the same ST was found in the milk filter as in milk samples collected 4–5 years earlier. Among the eight STs that had this pattern, ST99, ST100, and ST570 were found in two farms each, while the other five STs were found in one farm each. The ST99, ST100, and ST570 were further analyzed using cgMLST, and the number of allelic differences (AD) between isolates was visualized with a minimum spanning tree (Supplementary Figure 3). The cgMLST results showed a higher degree of relatedness among isolates originating from the same farm, including those collected in different years. These isolates were either identical or exhibiting fewer ADs compared to isolates from other farms.

Statistical analyses of the STs with at least five milk isolates (ST93, ST99, ST100, ST570, ST996, ST997) showed no significant association between ST and farm or county. Likewise, no specific patterns were found in the network analyses of cattle movements (data not shown). ST996 had significantly higher odds to be associated with persistent IMI than other STs (*p* < 0.05, OR = 24.7 95% CI = 1.1, 626.3). However, the analysis included only three ST996 isolates, two of which were from cows with persistent IMI. No significant associations were found between ST and CMT score.

#### 3.1.2 Genetic diversity of human *S. epidermidis*

Among the 100 isolates obtained from humans, 27 different STs were identified. Thirteen isolates from cattle farmers/personnel in 12 farms yielded 12 STs, including two newly identified ones (Table 1). Ten STs were exclusive to the farmers/personnel, while ST5 and ST59 were also detected in patients. The remaining 87 human isolates were predominantly associated with post-operative infections and originated from 35 patients. These isolates represented 17 STs, with ST2 and ST215 being the most prevalent, constituting 64% (56/87) of the isolates. The ST2 isolates (*n* = 32) were obtained from 13 patients, with the majority (8 isolates) from patient 2, followed by five isolates from patient 43, three isolates each from patients 37 and 56, and two isolates each from patients 12, 28, 47, and 58. Additionally, one ST2 isolate was recovered from each of the patients SE7 through SE11. Regarding the ST215 isolates (*n* = 24), they originated from 15 patients: 7 isolates from patient 1, 4 isolates from patient 40, and one isolate each from patients 37, 50, and SE12 to SE23. In four patients (28, 42, 43, and 58), isolates were obtained with intervals ranging from 13 to 508 days. Genotypic variations were identified in all isolates obtained from each patient who contributed multiple samples (Figure 4). The distribution of STs remained consistent over time between 1995 and 2011, with 68 isolates originating from Umeå University Hospital, and 19 from Östersund Hospital. The DI was higher among farmer isolates {DI = 0.987 [CI (5–95) = 0.956–1.0]; 12 STs/13 isolates} than among patient isolates {DI = 0.783 [CI (5–95) = 0.722–0.844]; 17 STs/87 isolates}.

#### 3.1.3 Comparison of bovine and human *S. epidermidis*

**Figure 5:**
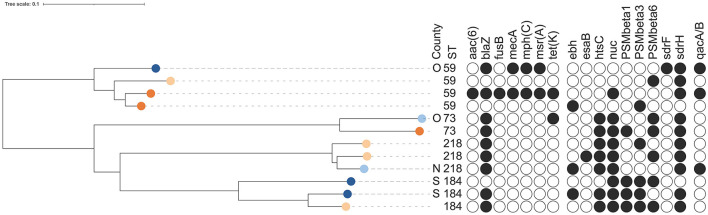
Dendrogram of core-genome multi-locus sequence typing of 12 isolates of *Staphylococcus epidermidis* belonging to sequence types (ST) present in both bovine and human isolates [three bovine milk isolates (dark blue), two bovine milk filter isolates (light blue), four isolates from healthy farmers/personnel (light orange) and three isolates from human hospital patients (dark orange)]. Information on sequence types, presence of antimicrobial resistance genes or point mutations, and detection of genes for potential virulence factors that were present in 5%−95% of all isolates (see Supplementary Table 1 for key) are shown. The scale is a unit-less ratio of core-genome multi-locus sequence typing allele similarities.

Four STs, ST59 (*n* = 4 isolates), ST73 (*n* = 2 isolates), ST184 (*n* = 3 isolates), and ST218 (*n* = 3 isolates), constituting 4.2% of all isolates (12 out of 283), were found among both bovine and human isolates (Table 1, Figure 5, Supplementary Figure 2). ST59 was found in samples from bovine milk, dairy farmers/personnel, and human patients, ST73 in samples from milk filters and patients, ST184 in samples from milk and farmers/personnel while ST218 was found in milk filters and farmers/personnel. In both the cgMLST and core-genome analyses for ST73 and ST128, the samples from a farmer and those from milk or a milk filter were genetically closer to each other than those from the same source (Figure 5, Supplementary Figure 2). Also, the pattern of gene absence and presence of the pan-genome largely followed the phylogeny of the samples rather than the sample type (Supplementary Figure 2).

In two (farms 10 and 25) of 17 farms contributing both bovine and human samples, *S. epidermidis* isolates were identified in both types of samples. One farm (farm 25) had one milk isolate and one nasal isolate from farmers/personnel, while the other farm (farm 10) had three milk isolates, five milk filter isolates and two nasal isolates from farmers/personnel. In none of the farms the same ST was found in both human and bovine isolates.

### 3.2 Presence of genes associated with resistance in *S. epidermidis*

Overall, a total of 11 AMR genes were found among the isolates (Table 3). In addition, point mutations linked to AMR were identified in six chromosomal genes. The distribution of AMR genes and point mutations detected in 5% to 95% of the isolates are presented in Figures 3, 4. The most common AMR genes were *fosB*, coding for fosfomycin resistance, and *blaZ*, coding for beta-lactamase production. Among point mutations, *parB* mut was found in all isolates. The number of AMR genes and point mutations identified were as follows; nine and five in milk isolates, five and four in milk filter isolates, eight and one in farmer/personnel isolates, and 10 and six in patient isolates. For most of the AMR genes and point mutations, the proportions of positive isolates were numerically higher among human isolates than among bovine isolates, and numerically higher among isolates from human patients compared to farmers/personnel. The average number of AMR genes differed between sample types. Particularly, the isolates from patients had significantly higher number of AMR genes (average = 4.76) than other sample types (*p* < 0.0001). Farmers had the second highest number of AMR genes (average = 2.38). The isolates from milk and milk filters had the lowest numbers of AMR genes (1.64 in milk; 1.60 in milk filters). Similar findings were observed for point mutations. The isolates from patients had a significantly higher number of cases of point mutations conferring AMR (average = 3.37) compared to other sample types (average = 1.05; *p* < 0.0001). All isolates from farmers had only one point mutation, a *parB* mutation (Table 3). By ST, isolates of ST2, ST188, ST215, and ST434, all of which were recovered only from patients, had higher number of AMR genes and point mutations on average compared to other STs. When patient ID was added as a random effect, as some of the isolates for ST2 and ST215 originated from same patients, *erm(C)* (*p* < 0.01) and point mutations in *rpoB* (*p* < 0.05) and *parE* (*p* < 0.01) were significantly associated with ST2, while presence of *erm(C)* (*p* < 0.05), *fusB* (*p* < 0.001), *tetK* (*p* < 0.0001), and point mutation in *dfrC* (*p* < 0.01) was significantly linked with ST215. As for the STs found in both human and bovine isolates, all 12 isolates each had only one point mutation (*parB*) and didn't have more AMR genes than other STs on average. However, two of four ST59 isolates, each recovered from milk and patient had *mph(C)* and *msr(A)* (Figure 5), while *mph(C)* and *msr(A)* were observed in six and five isolates in total in the study, respectively. As can be seen in Figure 5, there were also some differences in presence of genes between isolates within ST59, ST73, and ST184, but not within ST218, found in both human and bovine isolates.

**Table 3:** Numbers (%) of isolates with antimicrobial resistance (AMR) genes and numbers (%) of isolates with at least one point mutation (mut) among *Staphylococcus epidermidis* isolates from bovine milk, bovine milk filters, healthy dairy farmers/personnel, and human hospital patients.

**AMR gene or Point mutation (mut)**	**Coding for resistance to**	**Milk isolates *N* = 128**	**Milk filter isolates *N* = 55**	**Farmer/personnel isolates *N* = 13**	**Patient isolates *N* = 87**	**All isolates *N* = 283**
*aac(6)*	Aminoglycoside	0 (0)	0 (0)	1 (8)	74 (85)	75 (26)
*aadD*	Aminoglycoside	0 (0)	0 (0)	0 (0)	12 (14)	12 (4)
*blaZ*	Beta-lactamase	56 (44)	22 (40)	12 (92)	65 (75)	156 (55)
*fosB*	Fosfomycin	130 (100)	60 (100)	13 (100)	79 (91)	282 (99)
*fusB*	Fusidic acid	3 (2)	1 (2)	1 (8)	34 (39)	39 (14)
*erm(C)*	Macrolide	1 (1)	0 (0)	0 (0)	50 (57)	51 (18)
*mph(C)*	Macrolide	1 (1)	0 (0)	1 (8)	4 (5)	6 (2)
*msr(A)*	Macrolide	1 (1)	0 (0)	1 (8)	3 (3)	5 (2)
*mecA*	Methicillin	2 (2)	0 (0)	1 (8)	69 (79)	72 (25)
*gyrA* mut	Quinolone	1 (1)^1^	1 (2)^1^	0 (0)	49 (56)^1, 2^	51 (18)
*parB* mut	Quinolone	128 (100)^3^	55 (100)^3^	13 (100)^3^	87 (100)^3^	283 (100)
*parC* mut	Quinolone	1 (1)^4^	1 (2)^4^	0 (0)	51 (59)^4 − 6^	53 (19)
*parE* mut	Quinolone	2 (2)^7, 8^	0 (0)	0 (0)	13 (15)^8, 9^	15 (5)
*rpoB* mut	Rifampicin	0 (0)	0 (0)	0 (0)	22 (25)^10 − 14^	22 (8)
*strpS194*	Streptomycin	15 (12)	8 (15)	0 (0)	0 (0)	23 (8)
*tet(K)*	Tetracycline	3 (2)	2 (4)	1 (8)	25 (28)	31 (11)
*dfrC* mut	Trimethoprim	1 (1)^15^	1 (2)^15^	0 (0)	54 (62)^15^	56 (20)

^1^ S84Y; ^2^ S84F; ^3^ G24A; ^4^ S80F; ^5^ S80A; ^6^ D84G; ^7^ D43N; ^8^ N404S, ^9^ D434V; ^10^ D471E; ^11^ I527M; ^12^ H481N; ^13^ S486F; ^14^ S486Y; ^15^ F99Y.

The beta-lactamase gene *mecA* was found in 69 (79.3%) of the human isolates and in at least one of the isolates from 33 (94.3%) of the human patients. It was also found in one (7.7%) of the farmer isolates and in two (1.6%) of the SCM isolates, from different farms (Figure 4). One of the SCM isolates came from farm 19 from where two other isolates were analyzed, lacking *mecA* (Figure 3). One of the SCM isolates with *mecA* shared ST with a patient isolate with *mecA* (Figure 5).

The *qac*A/B genes were uncommon among milk isolates (3.9%), milk filter isolates (1.8%), and farmer isolates (0%), but common (47.1%) among isolates from human patients. The insertion sequence 256 (IS256) was identified only in isolates from human patients and was found in 46 out of 87 (52.9%) samples.

### 3.3 Presence of potential virulence genes in *S. epidermidis*

Detailed results on prevalence of pVF in all the isolates, and in isolates by sample type (milk, milk filters, farmers/personnel, and patients) are presented in Supplementary Table 2 and Supplementary Figure 1. The distribution of pVF found in 5%−95% of the bovine and human isolates is found in Figures 3, 4, respectively. Overall, the presence of 196 pVFs were studied. Of those 196 pVFs, 46 were identified at least once among the 283 isolates analyzed. When including all 283 isolates, the mean number of pVFs per isolate was 23.8 pVFs (SD 2.2; range 19–31). Divided by type of isolate, the numbers of unique pVFs identified in isolates from milk, milk filters, farmers/personnel and patients were 42, 35, 29, and 39, respectively. The corresponding numbers for the mean number of pVFs per isolate in each type of isolate were 23.1 (SD 1.6; range 19–28), 23.4 (SD 1.6; range 20–28), 23.0 (SD 1.5; range 21–26), and 25.2 (SD 2.7 range 20–31), respectively. The mean number of pVFs was significantly (*p* < 0.0001) higher in isolates from patients (*n* = 87) compared to in other types of isolates (*n* = 196).

When examined by functional group (adherence, exoenzyme, host immune evasion, iron uptake and metabolism, toxin) using the Wilcoxon rank-sum test, a significantly higher mean number of pVFs for adherence was observed (*p* < 0.0001) in isolates from patients (6.9; *n* = 87) compared to in isolates from other sample types (5.0; *n* = 196). Also, pVFs related to toxin genes (i.e., all genes from hemolysin onwards in Supplementary Table 2) were significantly higher among patient isolates (*p* < 0.05), although the difference was small (2.9 vs. 2.6). Isolates from milk and milk filters had significantly higher number of pVFs for iron uptake and metabolism (*p* < 0.05) than the other sample types, but the difference was also small (range: 8.5–8.9). There was no difference between sample types regarding host immune evasion pVFs, for which all 283 isolates had only *arcA* and *capP*.

The proportions of isolates being positive for a specific pVF varied from 0% to 100%. Overall, most (77.6%; 152 out of 196) of the pVFs studied, including for example many of the adherence factors, most of the host immune factors and enterotoxins, and all exotoxins, were not detected or present in very low proportions (< 5%). In contrast, 18 (9.2%) of the pVFs, i.e., *atl, sasC, icaD, aur, sspA, sspB, geh, arcA, capP, htsA, htsB, sbnA, sfaA, sfaB, sfaC, sfaD, hlb*, and *etc*., were very common (>95%). Thus, substantial variation in presence within and between sample types was mainly observed for 17 (8.7%) pVFs. These pVFs were *bhp/sesD, ebh, icaA, icaB, icaC, icaR, sdrF, sdrG, sdrH, sesI, nuc, isdG, htsC, sirC, esaB, PSM*β*1, and PSM*β*3*.

When comparing the distribution of each pVF in bovine (*n* = 183) vs. human (*n* = 100) isolates, *bhp/sesD* was only present in bovine isolates (*p* < 0.0001) while other pVFs, i.e., *sdrF* (*p* < 0.0001), *sspA* (*p* < 0.05). *sspB* (*p* < 0.01), *htsC* (*p* < 0.0001), *sirC* (*p* < 0.05), and *esa*β (*p* < 0.01), were significantly more common in bovine isolates than in human isolates. On the other hand, *sesI* was only found in isolates from human patients (*p* < 0.0001) while *icaA* (*p* < 0.0001), *icaB* (*p* < 0.0001), *icaC* (*p* < 0.0001), *icaR* (*p* < 0.0001), *sdrH* (*p* < 0.05), *isdG* (*p* < 0.0001), and *PSM*β*3* (*p* < 0.001) were significantly more common in human than in bovine isolates. Notably, *icaA, icaB, icaC, icaR*, and *sesI* were only observed in isolates from patients, and *PSMbeta3* was found significantly more common in isolates from patients (*n* = 87) than from farmers (*n* = 13) when compared to each other (*p* < 0.05).

When the mean number of pVFs was compared between STs, ST2 (*n* = 32), ST54 (*n* = 3), and ST188 (*n* = 5) had higher numbers of pVFs compared to the other STs. Notably, all the isolates of these three STs had *icaA, icaB*, and *icaC*, which are pVFs related to adherence. *icaR* was observed in all five isolates of ST188 and 84.4% of ST2 (*n* = 32), but not in any of the ST54 isolates. Further analysis showed that the presence and absence of each pVF was associated with STs. For instance, all ST100 isolates (*n* = 28), which were recovered from milk (*n* = 23) and milk filters (*n* = 5), contained *sdrG, isdG, htsC, sspA*, and *sspB*, but lacked *nuc, sirC*, or *esaB*. In contrast, all isolates of ST99, derived from milk (*n* = 18) and milk filters (*n* = 6), contained *nuc, sspA*, and *sspB*, but lacked *isdG* or *esaB*. ST570 (*n* = 34), another prevalent ST among bovine samples, consistently showed the presence of *nuc, isdG, htsC, sspA*, and *sspB* and the absence of *sirC* and *esaB* in all isolates from milk (*n* = 22) and milk filters (*n* = 12). Similarly, all ST2 isolates (*n* = 32), which were recovered from patients, contained *icaA, icaB*, and *icaC*, while all ST215 isolates (*n* = 24), also exclusively from patients, contained *sesI, nuc*, and *isdG*, but lacked *icaA, icaB, icaC, icaR, bhp*, or *ebh*. Further analysis of the associations using mixed-effects logistic regression, with sample type and herd added as random effects, showed that ST99 and the presence of *sirC* (*p* < 0.0001) was significantly associated. Similarly, ST2 was significantly associated with a higher occurrence of *icaR* (*p* < 0.01), *sesI* (*p* < 0.05), and *PSMbeta1* (*p* < 0.05) when patient ID was added as a random effect.

There were no significant differences regarding the four STs found in both human and bovine isolates (*n* = 12), except that the presence of *sdrF* was significantly less common (*p* < 0.05) in these STs compared to other STs, while all of them had *sdrG*. There was no significant association between the number of pVFs and having a persistent IMI, nor between the number of pVFs and CMT scores in milk.

## 4 Discussion

### 4.1 Comparisons between bovine and human isolates

Overall, only four STs (ST59, ST73, ST184, and ST218) were identified in both bovine and human isolates, representing a very small (4.2%) proportion of all isolates. The presence of identical STs in both groups could indicate either transmission between animals and humans or a shared bacterial source. Based on the data from the present study, the risk for such scenarios appears to be small. Nonetheless, the presence of the same ST in samples from farmers and bovine milk was expected given the close contact between farmers and animals on dairy farm, as well as findings from previous studies on MRSA and *S. epidermidis* (Ericsson Unnerstad et al., 2018; Thorberg et al., 2006; Jaglic et al., 2010). However, the present study included relatively few isolates from dairy farmers/personnel.

The distinction between *S. epidermidis* strains linked to bovine mastitis and human post-operative infections is evident. ST99, ST100, and ST570 are predominantly found in bovine mastitis and are rarely encountered in human isolates (Lee et al., 2023). Conversely, ST2 and ST215 are prevalent among Swedish patients, but are very unusual among bovine isolates (Argudin et al., 2015b). Of the four STs shared between human and bovine isolates, ST59 is frequently reported in clinical human isolates and has been identified in bovine mastitis in Germany and ovine mastitis in Greece (Katsarou et al., 2021). ST184 and ST218 are uncommon in both human clinical isolates and animals. ST100, and to a lesser extent ST570, have been extensively identified in healthy pig populations in Belgium since 2015 (Argudin et al., 2015a,b). Additionally, ST100 have been implicated in cases of bovine mastitis in Germany and ovine mastitis in Greece (Katsarou et al., 2021). The spread of ST100 and ST570 has also been observed in pig farms in South Korea, with ST100 detected in pig farmers, indicating potential zoonotic transmission (Lee et al., 2023). In the study by Lee et al. (2023), 14 linezolid-resistant *S. epidermidis* isolates belonged to ST100 or ST570, which also exhibited high levels of resistance to other antimicrobials and zinc chloride. These findings raise concerns as they suggest that some lineages like ST100 and ST570 may be more host adaptable while more prone to acquire resistance.

As expected, based on previous studies on non-aureus staphylococci, presence of AMR genes was more common among *S. epidermidis* isolates from hospital patients (Monsen et al., 1999; Lee et al., 2018; Widerström et al., 2016; Månsson et al., 2021) than among isolates from presumed healthy humans, i.e., farmers/personnel, and bovine milk and milk filters. The *aac(6)* and *aadD* genes, coding for aminoglycoside resistance, and the *mecA* gene, coding for methicillin resistance, were almost exclusively identified in isolates from human patients. The same was the case for most of the point mutations conferring AMR. This further lends support to the notion that antibiotic resistance is more common in Sweden among human clinical settings than among dairy cattle. In the present study, we exclusively identified IS256 in isolates of human origin and this IS has previously been linked to more pathogenic *S. epidermidis* strains in human clinical settings (Månsson et al., 2021). In addition, human isolates were also more prone to carry the *qacA/B* genes compared to isolates of bovine origin, which might be due to differences in types of disinfectants used in the different settings. Besides *blaZ*, the *fosB* was frequent in all STs from both human and bovine sources. The reason for this occurrence is unknown as fosfomycin is an old antibiotic not extensively used, but the results of this study correspond to results from previous studies on *Staphylococcus s*pp. (Lee et al., 2018; Kellgren et al., 2024). Interestingly, a limited number of isolates belonging to ST59 were identified both among bovine and human isolates, and these ST59 were associated with the carriage of both the genes *mph(C)* and *msr(A)*. The association between these genes is not unexpected, as it has been indicated that the *msr(A)* gene is needed for the expression of *mph(C)* (Matsuoka et al., 2003). However, since the *mph(C)* has primarily been linked to reduced susceptibility to macrolides and as macrolides are not used in bovines in Sweden, the detection of these genes in the same ST isolated from both humans and bovines could indicate a human origin for the bovine isolates. This is further supported by the fact that ST59 isolates are commonly identified in human clinical settings both in Sweden and globally (Månsson et al., 2021). Still, further studies with larger sample size is warranted to investigate the spread of ST59 among the bovine population and the epidemiological association with those from humans.

There were also some differences in prevalence of pVFs between bovine and human isolates, most notably that the gene *bhp/sesD* was only found in bovine isolates. However, according to Ortega-Pena et al. (2019) *sesD* is one of genes coding for surface proteins that was mainly seen in commensal isolates from healthy human skin rather than in isolates from prosthetic joint infections. *SesI* was predominately detected in isolates from patients and in the sequence types that were previously described as multidrug-resistant healthcare-associated sequence types with international spread (ST2 and ST215). The cell-wall-associated surface protein *SesI* has been shown to be associated with invasive isolates of *S. epidermidis* and is rarely found among colonizing isolates (Månsson et al., 2021).

### 4.2 Distribution and spread of bovine genotypes

Analyses of milk and milk filter isolates revealed significant genetic variation among bovine *S. epidermidis* isolates. However, the most common STs in both materials were ST99, ST100, and ST570. To our knowledge, few studies on bovine milk isolates have been published. Katsarou et al. (2021) reported that ST59 and ST91 were the most common among bovine milk isolates. However, the same authors also reported that ST100 and ST570, as well as ST59, have been identified as causative agents of ovine mastitis in Greece. ST100 and ST570 have also been identified in healthy veal calves and pig farms in Belgium and South Korea (Argudin et al., 2015a,b; Lee et al., 2023).

A number of STs identified in the current study, particularly the three most common STs, were found in multiple dairy farms from different regions, suggesting the possibility of inter-farm spread, potentially through cattle trade. However, the cgMLST analysis showed a larger allele difference for the ST99, ST100, and ST570 between farms than between isolates from the same farm, with isolates primarily grouping based on farm (Figure 3, Supplementary Figure 3). These results indicated that overall, no recent transmission between farms appears to have occurred. This conclusion is further supported by the fact that no association between genotypes, ST99, ST100, and ST570, and location of farms or cattle movements between participating farms could be established during the study period. The cgMLST results do, however, suggest that within-farm transmission has occurred, likely from a common source, such as during milking. Some allele was also observed within STs on individual farms, which may indicate that the STs were introduced on several occasions and/or from multiple sources. Similar findings based on cgMLST were reported for *S. chromogenes* in our previous study (Persson Waller et al., 2023). Additionally, the milk filter results demonstrated that the same STs, with similar cgMLST profiles, can persist within a farm for several years, indicating that the species is well-established in these farm environments. Similar within-farm persistence of *S. aureus* milk isolates has also been found previously (Anderson and Lyman, 2006). Persistence of *S. epidermidis*, defined as finding the same phenotypic species in two milk samples taken from the same udder quarter ~1 month apart, was found in 30% of the quarter cases reported by Nyman et al. (2018). In the present study, using milk isolates from the same study, we found that ST996 had higher odds of being associated with such persistent IMI than other STs. The two isolates were recovered from two different farms. However, this needs to be interpreted with caution as it was only 7 samples in total that were associated with persistent infection in the study and 2 of them had ST996, warranting further investigation. Although Nyman et al. (2018) found elevated milk CMT in quarters infected with *S. epidermidis*, we did not find any significant differences in inflammatory response as measured by milk CMT between STs in the present study. In our previous study on *S. chromogenes* and *S. simulans* we found differences in both persistency and CMT between clusters and STs for *S. chromogenes* but not for *S. simulans* (Persson Waller et al., 2023).

#### 4.2.1 Presence of antimicrobial resistance genes

Excluding *fosB*, the *blaZ* gene, coding for resistance to beta-lactams, was the most common AMR gene found among bovine isolates in the present study and was detected in 43% of the milk and milk filter isolates. This is in line with previous Swedish studies on the prevalence of beta-lactamase production in *S. epidermidis* from bovine IMI (Persson Waller et al., 2011; Nyman et al., 2018). In comparison, a recent Canadian study using similar methodology found *blaZ* in as much as 80% of the milk isolates (Nobrega et al., 2018). Moreover, they found the *mecA* gene, encoding methicillin resistance, in 17% of the isolates which was markedly higher than in the present study (2%). That the *mecA* was uncommon in *S. epidermidis* from Swedish bovine IMI is also in accordance with previous studies showing that methicillin resistance among *Staphylococcus* spp., including MRSA, is uncommon among animals in Sweden (Swedres-Svarm, 2023). Likewise, the presence of the *tet(K)* gene, encoding resistance to tetracycline, was much more common among the Canadian bovine milk isolates (38%) than among the Swedish isolates (3%). In contrast, the *strpS194* gene, indicating resistance to streptomycin, was found among 13% of the Swedish isolates but this gene was not reported in the study by Nobrega et al. (2018). Variations in presence of AMR in bovine milk isolates across different countries or regions are expected due to differences in bacterial strains and use of antibiotics.

The presence of the *blaZ* gene makes penicillin unsuitable for the treatment of mastitis cases. In Sweden, benzyl penicillin has been the drug of choice for the treatment of mastitis associated with Gram-positive bacteria for many decades. Thus, active, and successful measures have been taken over the years to reduce the prevalence of beta-lactamase producing *S. aureus* associated with IMI. However, this study indicates that further work is necessary to reduce the prevalence of beta-lactamase-producing NAS, such as *S. epidermidis*. The relatively high prevalence of the *strpS194* gene was unexpected, given that the use of streptomycin for systemic treatment of bovine mastitis has been very low or absent for decades in Sweden (K. Persson Waller, data not shown). However, dihydrostreptomycin was commonly used in dry cow therapy in Sweden during the study period. Presence of the *strpS194* gene, although in a lower proportion, was also detected among *S. chromogenes* and *S. simulans* milk isolates in a recent study from the same time period (Persson Waller et al., 2023).

Overall, the ST100 might be of particular interest in the present study since it appears to have a propensity for acquiring AMR genes, with a high prevalence (86%) of *blaZ*, compared to ST99 (33%) and ST570 (9%) isolates. Moreover, when comparing the occurrence of the *strpS194* gene, the prevalences were 25% for ST100, 0% for ST99, and 9% for ST570. The reason for the difference in AMR gene occurrence between STs needs further studies.

#### 4.2.2 Presence of potential virulence genes

A large number of different pVFs were found among the bovine milk isolates. Most of the pVFs found belonged to functional groups of adherence, exoenzymes or iron uptake/metabolism while toxin genes were mostly uncommon. Presence of virulence genes may be associated with variation in inflammatory response, but in the present study, we found no significant associations between presence of pVF and persistent IMI or degree of inflammatory response in the udder as measured by the milk CMT, an indirect measurement of the milk somatic cell count. In the present study, however, all isolates came from cases of SCM. A comparison between isolates from SCM and isolates from cases of clinical mastitis might have given a different response as suggested by Naushad et al. (2019). Moreover, the presence of genes does not indicate the expression of virulence factors, and many other factors, such as host immune response, play a role in the development of mastitis.

To our knowledge, few studies have been published on pVF in *S. epidermidis* isolates from bovine IMI, and none of those included information on STs. However, Naushad et al. (2019) reported pVFs for 26 isolates of *S. epidermidis* isolated from IMI in Canadian dairy cows. Many of the pVF results were similar in the two studíes, but discrepancies were also found. For example, presence of the genes *ebp, capH, capM, lip, splF*, and *uafA* was common among the Canadian isolates, but these genes were not found among the Swedish isolates. Moreover, the proportions of isolates with the genes *ebh, icaA, icaC, sdrF, sdrG, sdrH, isdG, htsC, sirB, PSM*β*1, PSM*β*2*, and *PSM*β*3* were numerically much lower in the present study than in the previous study (Naushad et al., 2019). In contrast, the proportions of isolates with the genes *icaD* and *sirC* were numerically much higher in the present study. In addition, the genes *arcA* and, *etc*. were commonly found among the Swedish isolates but was not reported in the Canadian study (Naushad et al., 2019). Differences between studies are probably due to regional variations of strains within species.

As mentioned, this is to our knowledge the first study evaluating differences in pVF between *S. epidermidis* STs. Although the low number of isolates per ST limited some statistical analyses and requires cautious interpretation, the results still indicated notable differences in the presence and absence of pVFs between STs. While the dataset size restricted the number of statistical analyses, the observed differences in pVF distribution among STs were evident. For example, when comparing the three most common STs (ST99, ST100, ST570), we found that the gene *isdG* was detected in all isolates of ST100 and ST570, but in none of the ST99 isolates. In contrast, the *nuc* gene was found in all ST99 and ST570 isolates, but in none of the ST100 isolates. Given the limited number of studies on this topic, further investigations are needed to examine the spread and clinical impact of specific phylogenetic lineages of *S. epidermidis* carrying different pVFs in bovine populations.

### 4.3 Methodological considerations

The comparison between human clinical isolates (1995–2011) and bovine milk isolates (2014–2015) should be interpreted with caution, as the collection periods for the bovine and human clinical isolates did not overlap (Figure 1). Additionally, the number of isolates obtained from healthy farmers/personnel was limited. For comparison, human *S. epidermidis* isolates were selected from two sources: first, consecutive clinical isolates from prosthetic joint infections, the majority of which were multidrug-resistant; and second, clinical isolates representing two previously described highly antibiotic-resistant hospital genetic lineages (ST2 and ST215). These isolates originated from two hospitals located within the same geographical region, ~400 kilometers apart. Although the number of bovine milk isolates included in the present study was larger than in a similar study (Naushad et al., 2019), the number of isolates per ST was mostly small making it difficult to evaluate differences in spread and presence of genes between types of *S. epidermidis*. Despite these limitations, we believe that the database is representable for Swedish bovine milk isolates and clinical human isolates. However, it is possible that the presence of specific genotypes may differ between regions and countries.

It is important to note that comparisons between results from different studies should be approached with caution, as differences in methodologies, such as database selection and the relatively understudied nature of *S. epidermidis*, may influence the findings. Moreover, identification of genes coding for AMR and pVFs indicate possible importance for infection and resistance. Their true relevance needs to be confirmed by phenotypic tests. Due to financial constraints, and the farm and cow data associated with the bovine *S. epidermidis* milk isolates (Nyman et al., 2018), we decided to focus our studies on between-farm and within-farm genotypic variation among isolates. Thus, we limited the number of isolates selected to one per farm and one per cow, respectively.

## 5 Conclusion

The study found limited overlap of STs and AMR genes between human and bovine isolates of *S. epidermidis*. However, the persistence of the same STs in multiple dairy farms suggests inter-farm spread. These findings provide new insights into the epidemiology of bovine *S. epidermidis* IMI and have implications for controlling such infections.

## Data Availability

The sequence data generated in this study has been deposited in the European Nucleotide Archive (ENA) at EMBL-EBI under accession number PRJEB79927 (https://www.ebi.ac.uk/ena/browser/view/PRJEB79927). The study also draws conclusions from data available in the SRA database under the BioProject accession number PRJNA557130 (https://www.ncbi.nlm.nih.gov/sra/PRJNA557130) and in the ENA BioProject accession number PRJEB44086 (https://www.ebi.ac.uk/ena/browser/view/PRJEB44086).
