# Supplementary material for: Dissemination of Staphylococcus epidermidis in Swedish bovine dairy herds: minimal overlap with human isolates
Source: Front Microbiol. 2025 Feb 7;16:1512461. doi: 10.3389/fmicb.2025.1512461 (PMC11849392; doi:10.3389/fmicb.2025.1512461)
Supplement: Supplementary file 1 [file Table_1.docx]

Supplementary Material

# Supplementary Tables

**Supplementary Table 1**. Command line arguments for bioinformatics programs used for analyses of the genomes of bovine milk isolates of *Staphylococcus epidermidis*

| Program | Arguments |
| --- | --- |
| Bowtie2 | Bowtie2-build --very-sensitive-local  Bowtie2 --phred33 --very-sensitive-local --no-unal |
| Pilon | Defaults |
| Prokka | --addgenes --genus Staphylococcus --species epidermidis --kingdom Bacteria  --proteins references/GCF_000007645.1_genomic_Sepidermidis.gbff |
| Roary | -e -i 90 |
| SPAdes | --careful |
| Trimmomatic | PE -phred33 ILLUMINACLIP:$illum_adaptors_path:2:30:5:1:TRUE LEADING:3 TRAILING:3 MAXINFO:40:0.2 MINLEN:36 |

**Supplementary Table 2**. Distribution (%) of potential virulence factors (pVF) among 283 isolates of *Staphylococcus epidermidis* from bovine milk (128 isolates), bovine milk filters (55 isolates), healthy dairy farmers/personnel (13 isolates) and human hospital patients (87 isolates)

| Function  groups | Virulence factors | Genes | Milk | Milk filter | Farmers/  personnel | Patients | All |
| --- | --- | --- | --- | --- | --- | --- | --- |
| Adherence | Accumulation- associated protein | *aap* | 0 | 0 | 0 | 0 | 0 |
|  | Autolysin | *atl* | 100 | 100 | 100 | 100 | 100 |
|  | Biofilm-associated surface protein | *bap* | 0 | 0 | 0 | 0 | 0 |
|  | Biofilm-associated protein (bap) homologue | *bhp/sesD* | 21.1 | 1.8 | 0 | 0 | 9.9 |
|  | Clumping factor A | *clfA* | 0 | 0 | 0 | 0 | 0 |
|  | Clumping factor B | *clfB* | 0 | 0 | 0 | 0 | 0 |
|  | Collagen adhesion | *can* | 0 | 0 | 0 | 0 | 0 |
|  | Elastin binding protein | *ebpS* | 0 | 0 | 0 | 0 | 0 |
|  | Cell wall associated fibronectin binding protein | *ebh* | 10.2 | 18.2 | 0 | 10.3 | 11.3 |
|  | Cell wall associated fibronectin binding protein | *efb* | 0 | 0 | 0 | 0 | 0 |
|  |  | *uafA* | 0 | 0 | 0 | 0 | 0 |
|  | Fibronectin binding protein | *fnbA* | 0 | 0 | 0 | 0 | 0 |
|  |  | *fnbB* | 0 | 0 | 0 | 0 | 0 |
|  | Formate dehydrogenase | *fdh* | 1.6 | 0 | 0 | 2.3 | 1.4 |
|  | Extracellular matrix binding protein | *embp* | 0 | 0 | 0 | 0 | 0 |
|  | Extracellular adherence protein/MHC analogous protein | *eap/ map* | 0 | 0 | 0 | 0 | 0 |
|  | Cell wall surface anchor family protein | *sasC* | 96.1 | 100 | 100 | 94.1 | 96.5 |
|  |  | *sasG* | 0 | 0 | 0 | 0 | 0 |
|  |  | *sraP* | 0 | 0 | 0 | 0 | 0 |
|  | Intercellular adhesin | *icaA* | 4.7 | 1.8 | 0 | 47.1 | 17.0 |
|  |  | *icaB* | 4.7 | 1.8 | 0 | 47.1 | 17.0 |
|  |  | *icaC* | 4.7 | 1.8 | 0 | 47.1 | 17.0 |
|  |  | *icaD* | 98.4 | 100 | 100 | 98.9 | 98.9 |
|  |  | *icaR* | 4.7 | 1.8 | 0 | 40.2 | 14.8 |
|  | Ser-Asp rich fibrinogen-binding protein | *sdrC* | 0 | 0 | 0 | 1.1 | 0.3 |
|  |  | *sdrD* | 0 | 0 | 0 | 0 | 0 |
|  |  | *sdrE* | 0 | 0 | 0 | 0 | 0 |
|  |  | *sdrF* | 54.7 | 56.4 | 38.5 | 21.8 | 44.2 |
|  |  | *sdrG* | 48.4 | 40.0 | 76.9 | 41.4 | 45.9 |
|  |  | *sdrH* | 49.2 | 72.7 | 84.6 | 67.8 | 61.1 |
|  |  | *sdrI* | 0 | 0 | 0 | 0 | 0 |
|  | *S. epidermidis* surface protein I | *sesI* | 0 | 0 | 0 | 69.0 | 21.2 |
| Exoenzymes | Adenosine synthase A | *adsA* | 0 | 0 | 0 | 0 | 0 |
|  | Aureolysin | *aur* | 100 | 100 | 100 | 100 | 100 |
|  | Cysteine protease (Staphopain) | *sspA* | 100 | 100 | 100 | 95.4 | 98.6 |
|  | Cysteine protease (Staphopain) | *sspB* | 100 | 100 | 100 | 90.8 | 96.8 |
|  |  | *sspC* | 0 | 0 | 0 | 0 | 0 |
|  |  | *sspD* | 0 | 0 | 0 | 0 | 0 |
|  |  | *sspE* | 0 | 0 | 0 | 0 | 0 |
|  |  | *sspF* | 0 | 0 | 0 | 0 | 0 |
|  | Hyaluronate lyase | *hysA* | 0 | 0 | 0 | 0 | 0 |
|  | Lipase | *lip* | 0 | 0 | 0 | 0 | 0 |
|  |  | *geh* | 100 | 100 | 100 | 100 | 100 |
|  | Serine protease | *splA* | 0 | 0 | 0 | 0 | 0 |
|  |  | *splB* | 0 | 0 | 0 | 0 | 0 |
|  |  | *splC* | 0 | 0 | 0 | 0 | 0 |
|  |  | *splD* | 0 | 0 | 0 | 0 | 0 |
|  |  | *splE* | 0 | 0 | 0 | 0 | 0 |
|  |  | *splF* | 0 | 0 | 0 | 0 | 0 |
|  | Staphylocoagulase | *coa* | 0 | 0 | 0 | 0 | 0 |
|  | Staphylokinase | *sak* | 0 | 0 | 0 | 0 | 0 |
|  | Thermonuclease | *nuc* | 75.8 | 90.9 | 84.6 | 89.7 | 84.8 |
|  | Von Willebrand factor-binding protein | *vWbp* | 0 | 0 | 0 | 0 | 0 |
| Host immune evasion | Arginine catabolic metabolic element | *arcA* | 100 | 100 | 100 | 100 | 100 |
|  | Capsule | *capA* | 0 | 0 | 0 | 0 | 0 |
|  |  | *capB* | 0 | 0 | 0 | 0 | 0 |
|  |  | *capC* | 0 | 0 | 0 | 0 | 0 |
|  |  | *capD* | 0 | 0 | 0 | 0 | 0 |
|  |  | *capE* | 0 | 0 | 0 | 0 | 0 |
|  |  | *capF* | 0 | 0 | 0 | 0 | 0 |
|  |  | *capG* | 0 | 0 | 0 | 0 | 0 |
|  |  | *capH* | 0 | 0 | 0 | 0 | 0 |
|  |  | *capI* | 0 | 0 | 0 | 0 | 0 |
|  |  | *capJ* | 0 | 0 | 0 | 0 | 0 |
|  |  | *capK* | 0 | 0 | 0 | 0 | 0 |
|  |  | *capL* | 0 | 0 | 0 | 0 | 0 |
|  |  | *capM* | 0 | 0 | 0 | 0 | 0 |
|  |  | *capN* | 0 | 0 | 0 | 0 | 0 |
|  |  | *capO* | 0 | 0 | 0 | 0 | 0 |
|  |  | *capP* | 100 | 100 | 100 | 100 | 100 |
|  | Chemotaxis inhibitory protein of *Staphylococcus* | *chp* | 0 | 0 | 0 | 0 | 0 |
|  | Staphylococcal complement inhibitor | *scn* | 0 | 0 | 0 | 0 | 0 |
|  | Staphylococcal protein A | *spa* | 0 | 0 | 0 | 0 | 0 |
|  | Staphylococcal binder of immunoglobulin | *sbi* | 0 | 0 | 0 | 0 | 0 |
| Iron uptake and metabolism | Iron-regulated surface determinant | *isdA* | 0 | 0 | 0 | 0 | 0 |
|  |  | *isdB* | 0 | 0 | 0 | 0 | 0 |
|  |  | *isdC* | 0 | 0 | 0 | 0 | 0 |
|  |  | *isdD* | 0 | 0 | 0 | 0 | 0 |
|  |  | *isdE* | 0 | 0 | 0 | 0 | 0 |
|  |  | *isdF* | 0 | 0 | 0 | 0 | 0 |
|  |  | *isdG* | 78.1 | 76.4 | 100 | 98.9 | 85.2 |
|  |  | *isdH* | 0 | 0 | 0 | 0 | 0 |
|  |  | *isdI* | 0 | 0 | 0 | 0 | 0 |
|  | ABS transporters (siderophore receptors) | *htsA* | 100 | 100 | 100 | 100 | 100 |
|  |  | *htsB* | 100 | 100 | 100 | 100 | 100 |
|  |  | *htsC* | 67.2 | 74.5 | 38.5 | 46.0 | 60.8 |
|  | NPQTN-specific sortase B | *srtB* | 0 | 0 | 0 | 0 | 0 |
|  | Staphyloferrin B synthesis-related | *sbnA* | 100 | 100 | 100 | 100 | 100 |
|  |  | *sbnB* | 0 | 0 | 0 | 0 | 0 |
|  |  | *sbnC* | 0 | 0 | 0 | 0 | 0 |
|  |  | *sbnD* | 0 | 0 | 0 | 0 | 0 |
|  |  | *sbnE* | 0 | 0 | 0 | 0 | 0 |
|  |  | *sbnF* | 0 | 0 | 0 | 0 | 0 |
|  |  | *sbnG* | 0 | 0 | 0 | 0 | 0 |
|  |  | *sbnH* | 0 | 0 | 0 | 0 | 0 |
|  |  | *sbnI* | 0 | 0 | 0 | 0 | 0 |
|  | ABC transporters (siderophore receptors) | *sfaA* | 100 | 100 | 100 | 100 | 100 |
|  |  | *sfaB* | 100 | 100 | 100 | 100 | 100 |
|  |  | *sfaC* | 100 | 100 | 100 | 100 | 100 |
|  |  | *sfaD* | 100 | 100 | 100 | 100 | 100 |
|  | Staphyloferrin A synthesis-related | *sirA* | 0 | 0 | 0 | 0 | 0 |
|  |  | *sirB* | 2.3 | 0 | 0 | 0 | 1.1 |
|  |  | *sirC* | 29.7 | 40.0 | 7.7 | 20.7 | 27.9 |
| Secretion system | Type VII secretion system | *esaA* | 1.6 | 0 | 0 | 0 | 0.7 |
|  |  | *esaB* | 10.2 | 20.0 | 7.7 | 1.1 | 9.2 |
|  |  | *esaC* | 0 | 0 | 0 | 0 | 0 |
|  |  | *essA* | 2.3 | 0 | 0 | 0 | 1.1 |
|  |  | *essB* | 1.1 | 0 | 0 | 0 | 0.7 |
|  |  | *essC* | 1.1 | 0 | 0 | 0 | 0.7 |
|  |  | *esxA* | 0 | 0 | 0 | 1.1 | 1.1 |
|  |  | *esxB* | 0 | 0 | 0 | 0 | 0 |
| Hemolysin | Alpha hemolysin | *hly/ hla* | 0 | 0 | 0 | 0 | 0 |
|  | Beta hemolysin | *hlb* | 100 | 100 | 100 | 100 | 100 |
|  | Delta hemolysin | *hld* | 0 | 0 | 0 | 0 | 0 |
|  | Gamma hemolysin | *hlgA* | 0 | 0 | 0 | 0 | 0 |
|  |  | *hlgB* | 0 | 0 | 0 | 0 | 0 |
|  |  | *hlgC* | 0 | 0 | 0 | 0 | 0 |
| Leukocidin | Leukocidin M | *lukM* | 0 | 0 | 0 | 0 | 0 |
|  |  | *lukF-like* | 0 | 0 | 0 | 0 | 0 |
| Leukotoxins | Panton-Valentine leukocidin | *lukS-PV* | 0 | 0 | 0 | 0 | 0 |
|  |  | *lukF-PV* | 0 | 0 | 0 | 0 | 0 |
|  | Leukotoxin D | *lukD* | 0 | 0 | 0 | 0 | 0 |
|  | Leukotoxin E | *lukE* | 0 | 0 | 0 | 0 | 0 |
| TSST | Toxic shock syndrome toxin | *tsst* | 0 | 0 | 0 | 0 | 0 |
| Exfoliate toxin | Exfoliate toxin type A | *eta* | 0 | 0 | 0 | 0 | 0 |
|  | Exfoliate toxin type B | *etb* | 0 | 0 | 0 | 0 | 0 |
|  | Exfoliate toxin type C | *etc* | 100 | 100 | 100 | 100 | 100 |
|  | Exfoliate toxin type D | *etd* | 0 | 0 | 0 | 0 | 0 |
| Phenol soluble modulins  (PSM) | PSM alpha | *PSMα1* | 0 | 0 | 0 | 0 | 0 |
|  |  | *PSMα2* | 0 | 0 | 0 | 0 | 0 |
|  |  | *PSMα3* | 0 | 0 | 0 | 0 | 0 |
|  |  | *PSMα4* | 0 | 0 | 0 | 0 | 0 |
|  |  | *PSMec* | 0 | 0 | 0 | 0 | 0 |
|  | PSM beta | *PSMβ1* | 11.7 | 1.8 | 7.7 | 24.1 | 15.9 |
|  |  | *PSMβ2* | 0 | 0 | 0 | 2.3 | 0.7 |
|  |  | *PSMβ3* | 25.0 | 30.9 | 15.4 | 54.0 | 34.6 |
|  |  | *PSMβ4* | 0 | 0 | 0 | 0 | 0 |
|  |  | *PSMβ5* | 0 | 0 | 0 | 0 | 0 |
|  |  | *PSMβ6* | 3.1 | 1.8 | 38.5 | 3.4 | 4.6 |
| Enterotoxin | Enterotoxin A | *sea* | 0 | 0 | 0 | 0 | 0 |
|  | Enterotoxin B | *seb* | 0 | 0 | 0 | 0 | 0 |
|  | Enterotoxin C | *sec* | 0 | 0 | 0 | 0 | 0 |
|  | Enterotoxin D | *sed* | 0 | 0 | 0 | 0 | 0 |
|  | Enterotoxin E | *see* | 0 | 0 | 0 | 0 | 0 |
|  | Enterotoxin G | *sef* | 0 | 0 | 0 | 0 | 0 |
|  | Enterotoxin H | *seg* | 0 | 0 | 0 | 0 | 0 |
|  | Enterotoxin I | *shi* | 0 | 0 | 0 | 0 | 0 |
|  | Enterotoxin J | *sej* | 0 | 0 | 0 | 0 | 0 |
|  | Enterotoxin-like K | *selk* | 0 | 0 | 0 | 0 | 0 |
|  | Enterotoxin-like L | *sell* | 0 | 0 | 0 | 0 | 0 |
|  | Enterotoxin-like M | *selm* | 0 | 0 | 0 | 0 | 0 |
|  | Enterotoxin-like N | *seln* | 0 | 0 | 0 | 0 | 0 |
|  | Enterotoxin-like O | *selo* | 0 | 0 | 0 | 0 | 0 |
|  | Enterotoxin-like P | *selp* | 0 | 0 | 0 | 0 | 0 |
|  | Enterotoxin-like Q | *selq* | 0 | 0 | 0 | 0 | 0 |
|  | Enterotoxin-like R | *selr* | 0 | 0 | 0 | 0 | 0 |
|  | Enterotoxin-like U | *selu* | 0 | 0 | 0 | 0 | 0 |
|  | Enterotoxin-like V | *selv* | 0 | 0 | 0 | 0 | 0 |
|  | Enterotoxin Yent1 | *yent1* | 0.8 | 0 | 0 | 0 | 0.3 |
|  | Enterotoxin Yent2 | *yent2* | 0 | 0 | 0 | 0 | 0 |
| Exotoxin | S. exotoxin 1 | *set1* | 0 | 0 | 0 | 0 | 0 |
|  | S. exotoxin 2 | *set2* | 0 | 0 | 0 | 0 | 0 |
|  | S. exotoxin 3 | *set3* | 0 | 0 | 0 | 0 | 0 |
|  | S. exotoxin 4 | *set4* | 0 | 0 | 0 | 0 | 0 |
|  | S. exotoxin 5 | *set5* | 0 | 0 | 0 | 0 | 0 |
|  | S. exotoxin 6 | *set6* | 0 | 0 | 0 | 0 | 0 |
|  | S. exotoxin 7 | *set7* | 0 | 0 | 0 | 0 | 0 |
|  | S. exotoxin 8 | *set8* | 0 | 0 | 0 | 0 | 0 |
|  | S. exotoxin 9 | *set9* | 0 | 0 | 0 | 0 | 0 |
|  | S. exotoxin 10 | *set10* | 0 | 0 | 0 | 0 | 0 |
|  | S. exotoxin 11 | *set11* | 0 | 0 | 0 | 0 | 0 |
|  | S. exotoxin 12 | *set12* | 0 | 0 | 0 | 0 | 0 |
|  | S. exotoxin 13 | *set13* | 0 | 0 | 0 | 0 | 0 |
|  | S. exotoxin 15 | *set15* | 0 | 0 | 0 | 0 | 0 |
|  | S. exotoxin 16 | *set16* | 0 | 0 | 0 | 0 | 0 |
|  | S. exotoxin 17 | *set17* | 0 | 0 | 0 | 0 | 0 |
|  | S. exotoxin 18 | *set18* | 0 | 0 | 0 | 0 | 0 |
|  | S. exotoxin 19 | *set19* | 0 | 0 | 0 | 0 | 0 |
|  | S. exotoxin 20 | *set20* | 0 | 0 | 0 | 0 | 0 |
|  | S. exotoxin 21 | *set21* | 0 | 0 | 0 | 0 | 0 |
|  | S. exotoxin 22 | *set22* | 0 | 0 | 0 | 0 | 0 |
|  | S. exotoxin 23 | *set23* | 0 | 0 | 0 | 0 | 0 |
|  | S. exotoxin 24 | *set24* | 0 | 0 | 0 | 0 | 0 |
|  | S. exotoxin 25 | *set25* | 0 | 0 | 0 | 0 | 0 |
|  | S. exotoxin 26 | *set26* | 0 | 0 | 0 | 0 | 0 |
|  | S. exotoxin 30 | *set30* | 0 | 0 | 0 | 0 | 0 |
|  | S. exotoxin 31 | *set31* | 0 | 0 | 0 | 0 | 0 |
|  | S. exotoxin 32 | *set32* | 0 | 0 | 0 | 0 | 0 |
|  | S. exotoxin 33 | *set33* | 0 | 0 | 0 | 0 | 0 |
|  | S. exotoxin 34 | *set34* | 0 | 0 | 0 | 0 | 0 |
|  | S. exotoxin 35 | *set35* | 0 | 0 | 0 | 0 | 0 |
|  | S. exotoxin 36 | *set36* | 0 | 0 | 0 | 0 | 0 |
|  | S. exotoxin 37 | *set37* | 0 | 0 | 0 | 0 | 0 |
|  | S. exotoxin 38 | *set38* | 0 | 0 | 0 | 0 | 0 |
|  | S. exotoxin 39 | *set39* | 0 | 0 | 0 | 0 | 0 |
|  | S. exotoxin 40 | *set40* | 0 | 0 | 0 | 0 | 0 |
| Mean (SD) numbers of pVF per isolate | | | 23.1 (1.6) | 23.4 (1.6) | 23.0  (1.5) | 25.2 (2.7) | 23.8 (2.2) |
| Min-max numbers of pVF per isolate | | | 19-28 | 20-28 | 21-26 | 20-31 | 19-31 |

## Supplementary Figures

**Supplementary Figure 1**. Dendrogram of core-genome multi-locus sequence typing of 283 bovine and human isolates of *Staphylococcus epidermidis* (128 milk isolates (dark blue), 55 bovine milk filter isolates (light blue), 13 isolates from healthy farmers/personnel (light orange), and 87 isolates from human hospital patients (dark orange). Information on sequence types (ST), presence of antimicrobial resistance genes or point mutations, and detection of genes for potential virulence factors that were present in 5 to 95% of all isolates (see Supplementary Table 1 for key) are shown. The scale is a unit-less ratio of core-genome multi-locus sequence typing allele similarities.

**Supplementary Figure 2**. Roary pangenome analysis of 12 isolates of *Staphylococcus epidermidis* belonging to sequence types (STs) present in both bovine and human isolates. The phylogenetic tree is based on the core genome alignment and gene presence/absence data across different isolates are visualized on the right in blue (presence) and white (absence), with the metadata (type, ST) in the middle.

**Supplementary Figure 3**. Minimum spanning tree constructed from 1840 cgMLST loci for ST99, ST100, and ST570. Isolates are color-coded when more than one isolate is included from the same farm (see Fig. 3).
**A. ST99**: Pink: farm 7; Purple: farm 15; Dark red: farm 17; Orange: farm 20; Yellow: farm 24.
**B. ST100**: Light blue: farm 2; Orange: farm 5; Pink: farm 7; Green: farm 9; Medium blue: farm 19.
**C. ST570**: Light blue: farm 2; Light green: farm 6; Dark pink: farm 10; Light pink: farm 11; Gray: farm 16; Teal: farm 21.

**
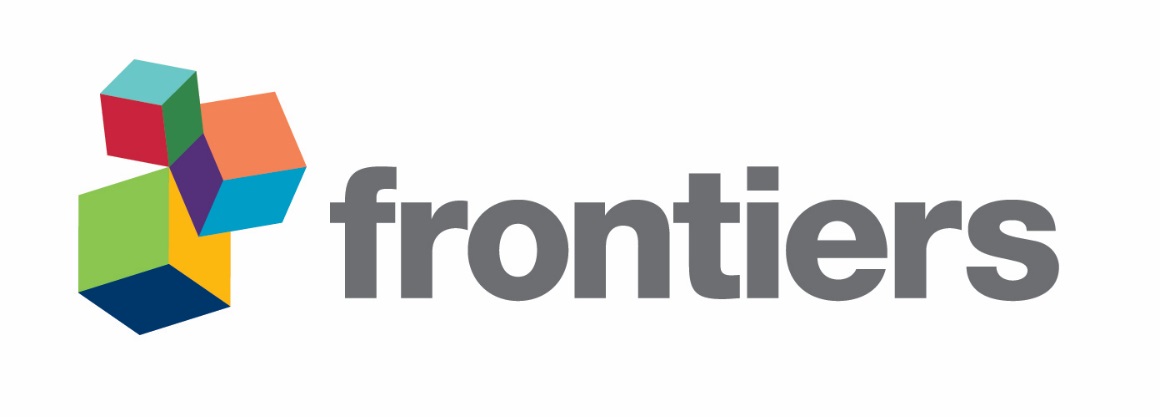
**

.
