# Supplementary figures and images for: Dissemination of Staphylococcus epidermidis in Swedish bovine dairy herds: minimal overlap with human isolates
Source: Front Microbiol. 2025 Feb 7;16:1512461. doi: 10.3389/fmicb.2025.1512461 (PMC11849392; doi:10.3389/fmicb.2025.1512461)

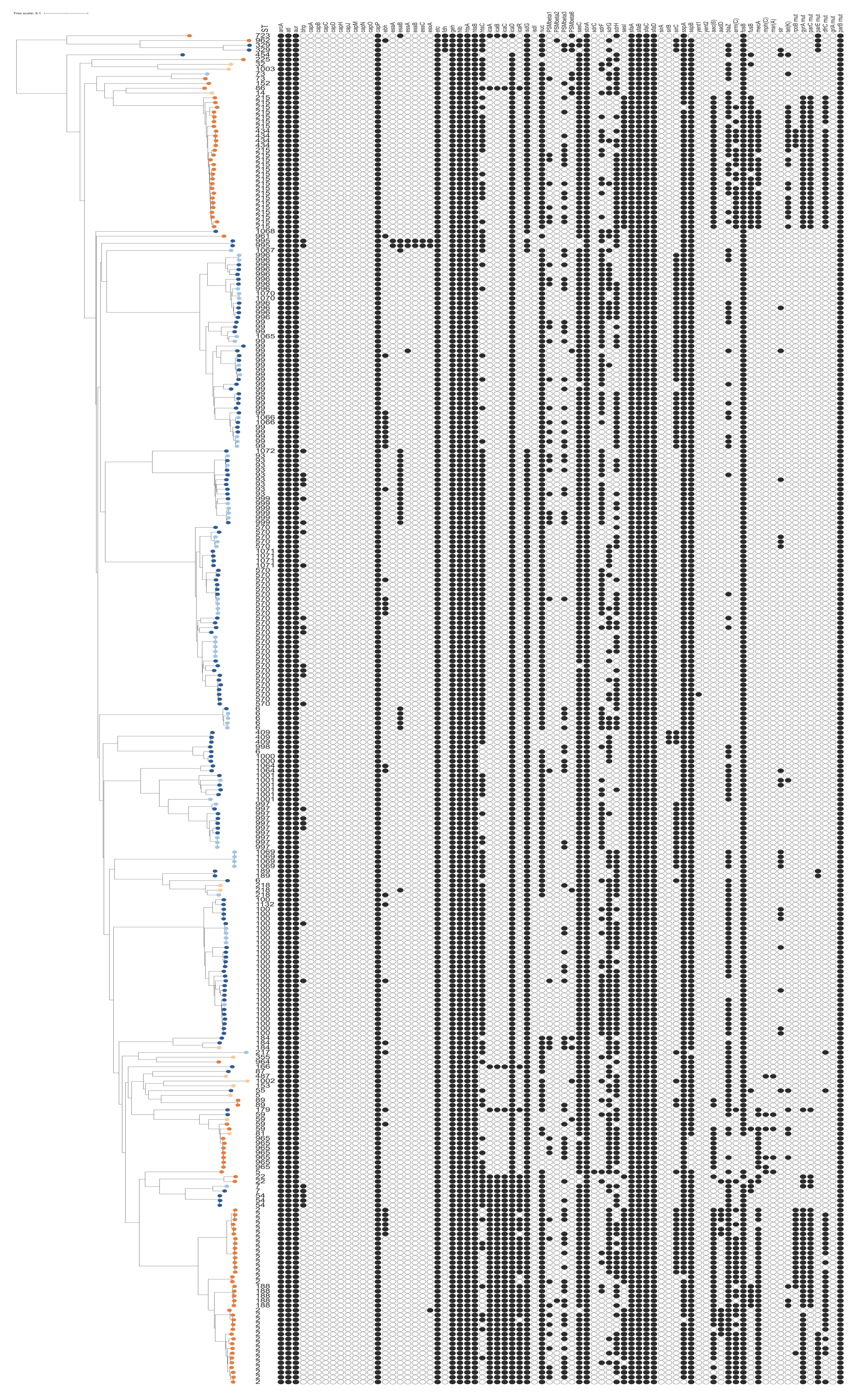

Supplement: Supplementary file 2 [file Image_1.jpg]

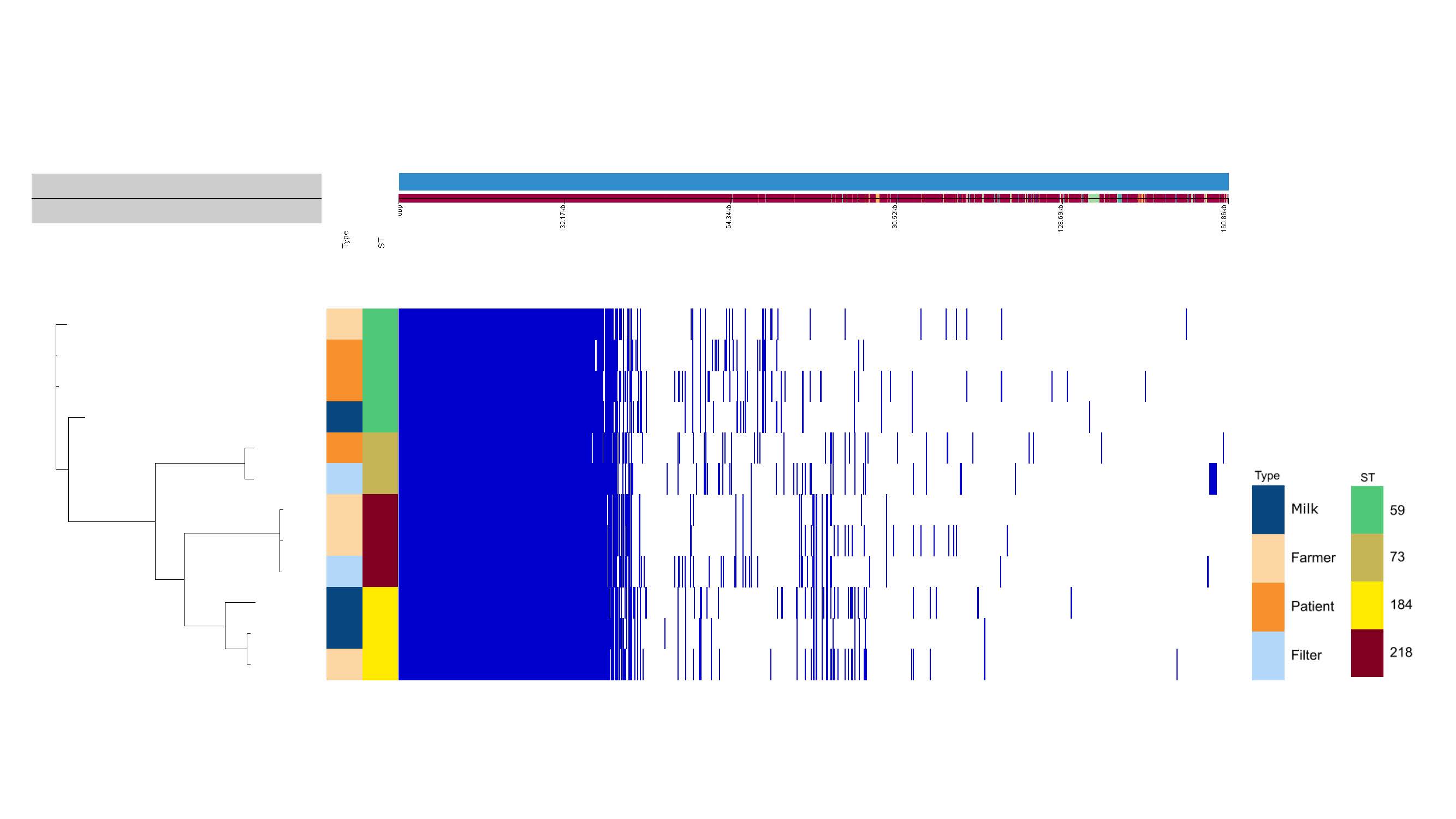

Supplement: Supplementary file 3 [file Image_2.jpg]

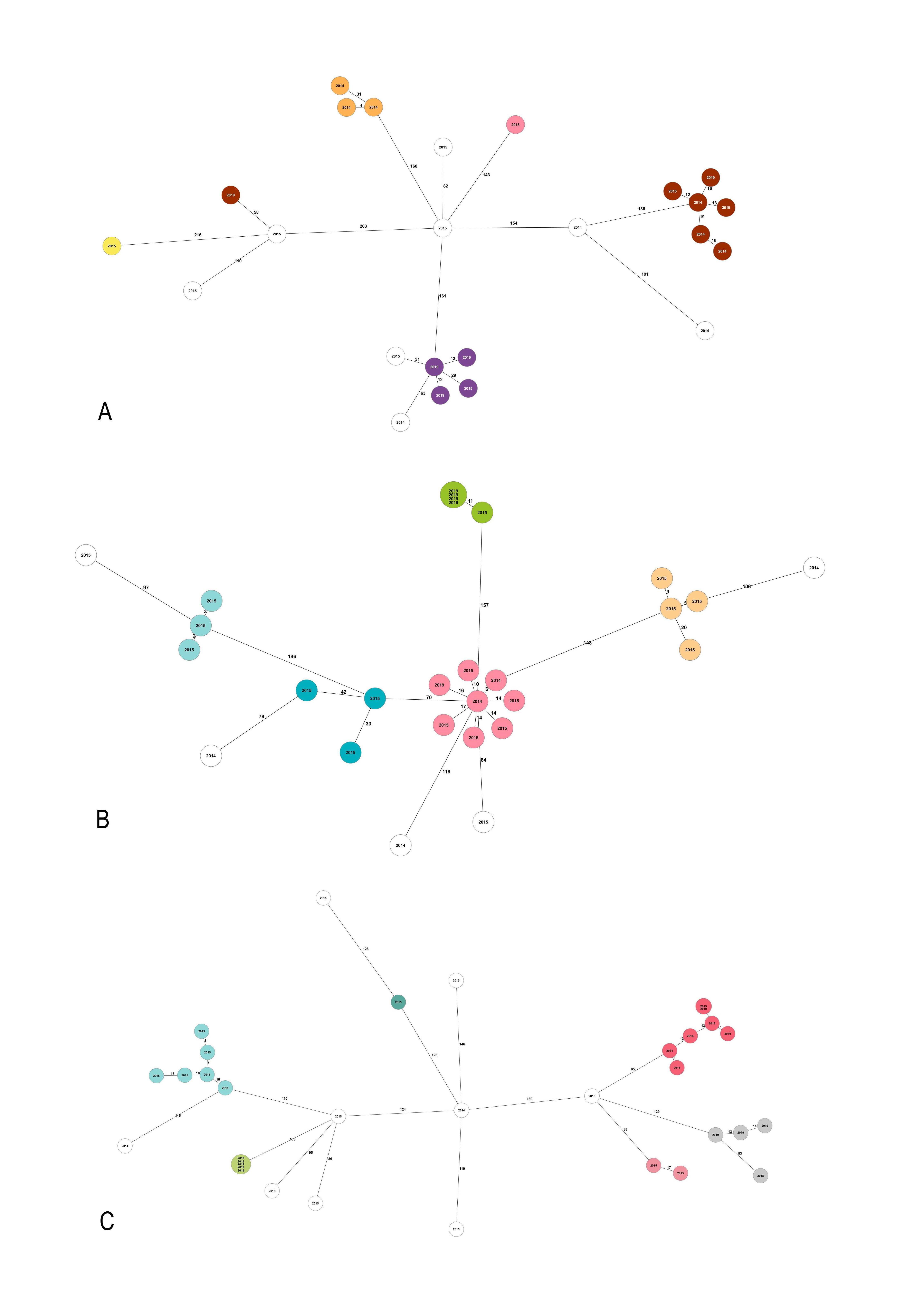

Supplement: Supplementary file 4 [file Image_3.jpg]
